# The origin and evolution of fibromelanosis in domesticated chickens: Genomic comparison of Indonesian Cemani and Chinese Silkie breeds

**DOI:** 10.1371/journal.pone.0173147

**Published:** 2017-04-05

**Authors:** Anik Budhi Dharmayanthi, Yohei Terai, Sri Sulandari, M. Syamsul Arifin Zein, Toyoko Akiyama, Yoko Satta

**Affiliations:** 1 Department of Evolutionary Studies of Biosystems, SOKENDAI (The Graduate University for Advanced Studies), Kanagawa, Japan; 2 Museum Zoologicum Bogoriense, Research Center for Biology, Indonesian Institute of Science (LIPI), Cibinong, Indonesia; 3 Department of Biology, Keio University, Yokohama, Japan; Charles University, CZECH REPUBLIC

## Abstract

Like Chinese Silkie, Indonesian Ayam Cemani exhibits fibromelanosis or dermal hyperpigmentation and possesses complex segmental duplications on chromosome 20 that involve the *endothelin 3* gene, *EDN3*. A genomic region, DR1 of 127 kb, together with another region, DR2 of 171 kb, was duplicated by unequal crossing over, accompanied by inversion of one DR2. Quantitative PCR and copy number variation analyses on the Cemani genome sequence confirmed the duplication of *EDN3*. These genetic arrangements are identical in Cemani and Silkie, indicating a single origin of the genetic cause of Fm. The two DR1s harbor two distinct *EDN3* haplotypes in a form of permanent heterozygosity, although they remain allelic in the ancestral Red Jungle Fowl population and some domesticated chicken breeds, with their allelic divergence time being as recent as 0.3 million years ago. In Cemani and Silkie breeds, artificial selection favoring the Fm phenotype has left an unambiguous record for selective sweep that extends in both directions from tandemly duplicated *EDN3* loci. This highly homozygous tract is different in length between Cemani and Silkie, reflecting their distinct breeding histories. It is estimated that the Fm phenotype came into existence at least 6600–9100 years ago, prior to domestication of Cemani and Silkie, and that throughout domestication there has been intense artificial selection with strength s > 50% in each breed.

## Introduction

With conspicuously diversified phenotypes with respect to morphological, physiological, and behavioral traits, domesticated animals are excellent model organisms for investigating underlying genetic changes as well as for elucidating the underlying evolutionary mechanisms. It is widely accepted that phenotypes currently observed in domesticated organisms are usually selected from variations that arose spontaneously in wild, ancestral populations. There have been several attempts to identify the genetic bases of such phenotypes and compare them with those of wild ancestors [1–5]. One of the common underlying ideas is that artificial selection reduces the level of genetic variability at linked neutral sites when a selected allele rapidly increases in frequency toward fixation (selective sweep) and/or is kept fixed in a breeding population for a relatively short period. On the basis of 2.8 million SNPs, the International Chicken Polymorphism Map Consortium [6] scanned the genomes of three chicken breeds (Broiler, Layer, and Silkie) and Red Jungle Fowl (RJF), the wild ancestor of domestic poultry. It was found that relatively high levels of genetic variation with nucleotide diversity *π* = 0.5% are maintained within chicken breeds; however, little evidence is provided for selective sweeps by adaptive (favored) alleles on length scales greater than 100 kb. One reason for the lack of such long-stretch signals could be the rather high recombination rate in chickens [7]. In a small-scale study of 32 introns randomly selected in two chicken breeds (Silkie and Koshamo or fighting cock), RJF and Green Jungle Fowl (GJF), Sawai et al. [8] also showed that domesticated chickens usually maintain nearly the same level of nucleotide diversity as their ancestral RJF population. The authors further argued that genomic regions that respond to domestication might be rather limited. However, re-sequencing of genomic DNA pools representing eight different populations of domesticated chickens and RJF demonstrated a number of regions under selective sweeps [9]. Another selective sweep analysis of feral Kauai chickens derived from domesticated populations identified genomic regions that are associated with comb mass, maternal brooding behavior and fecundity [10]. Unfortunately, however, these studies did not cover all interesting phenotypes of the various chicken breeds, including Silkie (S1 Fig).

In contrast to these genome-wide scan approaches, we took a candidate gene approach and focused on a particular phenotype known as fibromelanosis (Fm) or dermal hyperpigmentation [11]. Mutations of the *Fm* gene result in excessive accumulation of black pigment in the skin and several other tissues or organs such as blood vessels, muscles, gonads and tracheas. Chinese Silkie is one of the domesticated chicken breeds with the Fm. The phenotype is inherited in a Mendelian fashion with semidominance [12]. Recombinant analysis using Silkie and Black Minorca (a homozygote for the wild-type chromosome regarding *Fm*) located the genomic region of *Fm* between 10.2 Mb and 11.7 Mb on chromosome 20 [12, see also 13,14].

It has been established that the *Fm* mutation is positively correlated with the duplication of a segment that contains the *EDN3* gene encoding endothelin 3 [12,14,15]. EDN3 is a major controller of neural crest cell movement and proliferation. Neural crest cells are pluripotent and thus can develop into several cell types, such as melanoblasts [16–19]. Melanocytes, which differentiated from melanoblasts, produce eumelanin (black and dark pigment melanin) and phaeomelanin (colored melanin) in the skin, comb and other organs [20]. The amount of *EDN3* mRNA in whole Silkie embryos at 18 days is approximately twice as high as that in wild-type chicken embryos [12,14]. Thus, *EDN3* is the most likely candidate gene for such coloring phenotypes in Silkie as well as other domesticated animals, including cats [21] and cattle [22]. Indeed, PCR and next-generation sequencing (NGS) analyses of the Silkie genome unveiled segmental duplication in the *Fm* region [14,23]. Previously, Dorshorst et al. [14] showed that two regions (DR1 and DR2), separated by a 417 kb spacer, underwent inverted duplication. In the reference genome (Gallus_gallus_4.0, http://www.ncbi.nlm.nih.gov), DR1 is located at nt positions 11,111,559 to 11,238,796 and DR2 at positions 11,651,876 to 11,822,527 on chromosome 20. Each of the duplicated DR1s is 127 kb long, and contains not only *EDN3* but also *HIVEP3*, *SLMO2*, *ATP5E*, and *TUBB1*, whereas each of the duplicated DR2s is 171 kb long and is devoid of genes.

Dermal hyperpigmentation is found in other domesticated chicken breeds, such as Ayam Cemani in Indonesia (S1 Fig), Kadakhnath in India, Black H’Mong in Vietnam, Argentinean Tuzo type in Argentina, and Svarthöna in Sweden. While they all show excessive melanin accumulation, the overall phenotypes of Cemani and other black chickens differ greatly from those of Silkie [13,14]. For instance, unlike Silkie, which shows fluffy feathering, Cemani shows black plumage and non-fluffy feathering. Moreover, comb shape in Cemani males is very different from that in Silkie males with rose combs. In light of these similarities and differences between Cemani and Silkie, Shinomiya et al. [12] and Dorshorst et al. [14] examined whether the *Fm* region in Cemani is functionally and structurally the same as that in Silkie. Shinomiya et al. [12] analyzed the progenies of sib-crosses of F1 hybrids between Cemani and Ayam Arab (a wild-type domesticated breed in Indonesia). Based on the copy number variation (CNV) observed in *EDN3* by quantitative PCR (qPCR), they suggested *EDN3* duplication in Cemani, but not in Ayam Arab. Similarly, using a PCR-based diagnostic test, Dorshorst et al. [14] found that the complex arrangement of DR1 and DR2 is shared among Silkie, Cemani, Black H’Mong and Svarthöna. However, because the two copies of DR1 and DR2 cannot be easily distinguished from each other by PCR or NGS, the precise genomic arrangement of these four regions has not fully been elucidated even in Silkie.

In this study, we compared the genomic structure, the pattern and level of DNA variation, and the evolutionary history of the *Fm* region between Cemani and Silkie. We paid particular attention to the genomic signature of artificial selection on a target gene, *EDN3*, and used it to estimate the duration and strength of artificial phenotypic selection.

## Materials and methods

### Ethics statement

This study was conducted in accordance with the animal research guidelines of SOKENDAI (The Graduate University for Advanced Studies), Hayama, Japan. The Research Ethics Committee of SOKENDAI approved the research protocol No.46 on June 16, 2016. Chicken blood and DNA samples were provided by the Indonesian Institute of Science (LIPI). Blood was sampled according to the procedure of animal welfare of the Museum Zoologicum Bogoriense (MZB), Division of Zoology, Research Center for Biology, LIPI, Indonesia. DNA samples were also obtained from Keio University via the Avian Bioscience Research Center (ABRC) of Nagoya University in Japan. Bird maintenance and blood collection were performed in accordance with the University-institutional guidelines for animal experiments.

In addition, Silkie DNA samples from the USA were obtained from the Japanese Society for the Study of H. I. H. Prince Akishinonomiya Collections (JSAC).

### Chicken breeds and DNA samples

Most domestic chickens of Cemani, Silkie and other breeds as well as the two jungle fowl species (RJF and GJF) were collected at various locations in Indonesia (Table 1). Chickens for DNA isolation were collected at farms in rural areas of Java, Sumatra, Sulawesi and Nusa Tenggara, Indonesia, mainly in 2005–2010, although some were obtained in more recent years. During sample collections, we always carried a letter of assignment from LIPI; however, no explicit permission from the farmers was required as chickens are not protected animals in Indonesia.

**Table 1:** IDs of 75 sampled domesticated chickens and jungle fowl together with their collection sites and sample sizes.

No	Chicken breed	Sample ID	Collection site	*n*^d^
1	Ayam Cemani	Cemani 40–47^a^	Kedu, Central Java, Indonesia	13
		CM (1, 6, 11, 23, 31)^a^		
		Cemani^b^	Nagoya, Japan	1
2	Silkie	SIB (2, 6, 7, 11, 14, 15, 16, 17)^c^	Murray McMurray Hatchery, Iowa, USA	8
		WS (3741,BS3846)^b^	Japan	5
		SIL (7123, 7124, 9541)^b^		
		KPS (16, 17, 30)^a^	BPTU-Sembawa, Palembang-Sumatera	3
3	Ayam Arab (Silver)	ARS15^a^	BPTU Ayam, Sembawa, South Sumatera, Indonesia	1
	(Golden)	ARG19^a^		1
4	Ayam Kedu (Hitam)	KD (1–5, 7–17,19, 21, 22)^a^	Kedu, Temanggung, Central Java, Indonesia	21
		KDH (3,8)^a^		
5	White Kedu (Ayam Kedu Putih)	KDP (1, 5, 7)^a^	Kedu, Temanggung, Central Java, Indonesia	3
6	Ayam Kalosi	KAL (28, 7, 2) ^a^	Gowa, South Sulawesi, Indonesia	3
7	Ayam Kate	KT9^a^	Yogyakarta, DIY, Indonesia	1
8	Ayam Sentul	STC13 ^a^	Sentul, West Java, Indonesia	1
9	Kampung Chicken	LOM39 ^a^	Lombok, Indonesia	1
10	Black Minorca	BMC (610, 613)^b^	Japan	2
11	Red Jungle Fowl (RJF)*	BKL(1, 2)^a^	Bengkulu, Indonesia	2
		Aceh (1, 4)^a^	Nangroe Aceh Darusalam, Indonesia	2
		RJF (1, 9527)^b^	Nagoya, Japan	2
12	Green Jungle Fowl (GJF)	BYW (2, 4)^a^	Banyuwangi, East Java, Indonesia	2
		BOJA1^a^	Boja, Kendal, Indonesia	1
		Bd (72, 92)^a^	Sumbawa, West Nusa Tenggara, Indonesia	2
		FL9^a^	Flores, East Nusa Tenggara, Indonesia	1

* BKL1, BKL2, Aceh1 and Aceh4 are *Gallus gallus spadiceus*, while RJF1 and RJF9527 are *Gallus gallus* with unknown subspecies name:

^a^ Chicken breeds and genomic DNAs acquired from the MZB, LIPI in Indonesia:

^b^ Genomic DNA samples supplied from Keio University via ABRC of Nagoya University in Japan:

^c^ Genomic DNA samples provided through the JSAC:

^d^ The number of samples

### Construction of Cemani genomic sequence library

Total DNA of one Cemani chicken (sample “Cemani 41”) was sheared into ~500-bp fragments using an M220 focused ultrasonicator™ (Covaris) and a genomic library NGS was prepared in accordance with the True Seq DNA PCR-free sample preparation protocol. Another genomic DNA library was prepared in accordance with protocols provided with the Illumina Nextera X, for nine regions, each of about 3 kb in length with ~200-kb intervals. Each 3-kb region was amplified by primers designed in-house (S1 Table) in a 30 μl reaction mixture (see S2 Table for the reaction conditions of PCR1). PCR products of each sample (5 μl) were pooled and purified using Agencourt AMpure XP (Beckman Coulter). The libraries were qualitatively and quantitatively verified using an Agilent Bioanalyzer and sequenced on the Illumina HiSeq2000 platform (Illumina).

### Public data used

The chicken reference genome was downloaded in September 2015 from the UCSC Genome Browser (https://genome.ucsc.edu/) and has the same sequence as that deposited in the GenBank database (Gallus_gallus_4.0). Additionally, full data sets of Silkie (accession numbers: SRX286765, SRX286766, SRX286773, SRX286776, and SRX286777, see ref. 23) and Taiwanese L2 (accession numbers: SRX286779-SRX286781, SRX286798, and SRX286799, see ref. 23) were retrieved from GenBank (http://www.ncbi.nlm.nih.gov/).

### Amplification of duplication boundaries

The presence or absence of duplication boundaries was examined by PCR with two previously published primer pairs, A2 and B2 [14], on a 96-Well GeneAmp® PCR System 9700 from Applied Biosystems (see S2 Table for the detailed reaction conditions of PCR2).

### Quantification of gene copy numbers

PCR products for *EDN3* (113 bp, primer set qAS044) and *uridine-cytidine kinase 1-like 1* (*UCKL1*) (124 bp, primer set q46) were ligated to the pMD20 vector (TaKaRa Japan). Plasmid DNA was extracted using alkaline lysis [24] and the concentration was determined using NanoVue spectrophotometer (GE Healthcare). Plasmid DNAs were diluted to 10^−1^ to 10^−6^ ng/μl in distilled water and were used to draw a standard curve for quantification. qPCR for absolute quantitative analysis was carried out with the SYBR® Premix Ex Taq™ II (Tli RNaseH Plus; TaKaRa). All reactions were run in triplicate on a Thermal Cycler Dice (Applied Biosystems), and the thermal cycling conditions were as indicated under “PCR3” in S2 Table.

### Amplification and sequencing of *EDN3*

A ~1-kb genomic fragment encompassing exons 4 to 5 of *EDN3* was amplified and sequenced with the previously reported primer pair AS044F and AS044R [12]. PCR was conducted under reaction conditions listed under “PCR4” in S2 Table. Amplified PCR products were purified by isopropanol precipitation and directly sequenced. For heterozygous sequences, the PCR products were cloned into pMD20, and eight clones for each product were sequenced using the BigDye Terminator Cycle Sequencing Kit (Applied Biosystems) with M4 and Rv universal primers on an ABI 3130xl sequencer (Applied Biosystems).

### Reads of data from the chicken WGS and nine 3 kb regions, and CNV analysis of the Fm region

The CLC Genomic Workbench 8.0.3 (www.qiagenbioinformatics.com) was used to map the 3-kb region reads to the reference genome with 90% length and similarity fractions.

To analyze the WGS of Cemani, Silkie, and Taiwanese L2, low-quality bases were removed with the Trimmomatric software [25], using the following parameter settings; leading = 10, trailing = 10, sliding windows = 4:15, and minlen = 40. The Samtools workflow [26–30] (http://www.htslib.org/workflow/) was used for mapping of the WGS data with 30X coverage.

To examine CNV in the *Fm* region, the reads from each of three pairs among Cemani, Silkie, and L2 WGS data were mapped onto nt positions 10,700,000–12,000,000 on chromosome 20 using the CNV-Seq software [31]. Default parameters (log2-threshold = 0.6, *p*-value = 0.01, and minimum windows = 4) were used to produce the CNV list.

### Statistical and population genetics analyses

The DNA sequences were aligned with the ATGC software (GENETIX). The number of nucleotide differences per site (*p*-distance) was calculated with Molecular Evolutionary Genetics Analysis (MEGA6) [32]. Neighbor-joining (NJ) trees [33] were constructed with 1000 bootstrap resampling with an option of complete deletion of gaps/missing nucleotides. The ratio of the extent of divergence to that of polymorphism between any of the nine 3-kb regions was tested using the HKA test [34] implemented in DnaSP [35]. Genetic components in Cemani, Silkie, other domesticated breeds, RJF, and GJF were examined using STRUCTURE (version 2.2) analysis [36] on the http://pritch.bsd.uchicago.edu/software website. Heterozygosity at individual SNP sites was computed based on allele frequencies in the samples (S3 Fig).

Calculations of the allele age or the time span of artificial selection operation were based on the same idea used for adaptively introgressed tracts [37,38]. It was assumed that the probability of observing a tract length ≥ *x* follows the exponential distribution:

$$P\{tract\geq x\}=exp(-\frac{x}{L})$$ (1)

where *L* is the mean tract length. This *L* is given approximately by $L=\frac{1}{r^{*}t}$, in which *t* is the number of generations elapsed during artificial selection, *r* is the recombination rate between two adjacent sites per generation, and r^*^ = *r*(1 − *f*) is the recombination rate in the presence of inbreeding, with inbreeding coefficient *f*. If *L* is equated to an observed mean tract length $\hat{x}$, we have an estimate of $t=\frac{1}{r\hat{x}(1-f)}$.

To estimate the selection coefficient, we used the following formula for the expected nucleotide diversity (*π*) at linked neutral sites under recent selective sweep. The ratio of *π* to the diversity before the sweep (*π*_0_) is given by

$$\frac{\pi }{\pi_{0}}=1-e^{-\frac{2r^{*}x}{s}ln(2N_{e}s)}$$ (2)

where *s* is the selection intensity for mutant homozygotes and *N*_*e*_ is the effective population size [1,39, 40–43]. It is clear from the formula that the substantial reduction is expected only if 2r^*^x/*s* in the exponent is as small as 0.01 [1] or roughly *s* = 200r^*^x. We note that this estimate is almost independent of 2*N*_*e*_*s*, unless *N*_*e*_ is unlikely large.

### Data deposition and availability

The nucleotide sequence data was deposited into the DDBJ databank. The sequences of the nine 3-kb regions by NGS are accessiblee from URL of http://trace.ddbj.nig.ac.jp/DRASearch (accession number: DRA004942) and 1-kb sequences for *EDN3* gene from URL of http://getentry.ddbj.nig.ac.jp/ (accession numbers: LC194635-LC194778). Aligned sequences for both 1-kb and 3-kb regions are available upon request.

## Results

### Single origin of the Fm phenotype

As Cemani and Silkie exhibit the same Fm phenotype (S1 Fig), the chromosomal rearrangement including duplicated *EDN3* was suspected to be the common genetic cause of Fm in both breeds [12,14]. Therefore, we first confirmed that the genomic rearrangement is indeed of single origin and common to Cemani and Silkie.

First, we studied genetic variation at *EDN3* in nine chicken breeds—Ayam Cemani (*n* = 5), Silkie (*n* = 3), Ayam Arab (*n* = 2), Ayam Kedu (*n* = 5), White Kedu (*n* = 2), Ayam Kalosi (*n* = 2), Ayam Kate (*n* = 1), Ayam Sentul (*n* = 1), and Kampung Chicken from Lombok (*n* = 1), and two jungle fowl populations, RJF (*n* = 6) and GJF (*n* = 6) (Table 1). To obtain unambiguously phased genomic sequence data for possibly four different *EDN3* genes in certain individuals, we analyzed DNA sequences of about 1 kb in length spanning exons 4 to 5. We selected this rather short fragment to avoid any complication due to intragenic recombination in inferring ancestral relationships among the DNA sequences. Indeed when we used 3-kb sequences for determining the *EDN3* phylogeny, we found strong evidence for intragenic recombination in multiple haplotypes, though not in those of Cemani and Silkie for an obvious reason (see NJ tree of region 4 in S4 Fig). The 1-kb sequences obtained from the 36 individuals in our sample can be classified into 12 haplotypes. Of these, six (*Hap*2’, 5, 6, 8, 10, and 11) are restricted to the jungle fowls, three (*Hap*1, 3, and 9) are restricted to domesticated chickens, and the remaining haplotypes (*Hap*2, 4, and 7) occur in both domesticated chickens and jungle fowls (Table 2). RJF and GJF individuals exhibit a relatively large number of distinct haplotypes and maintain higher haplotype diversity than domesticated chickens. Importantly, however, no individual possesses more than two distinct haplotypes, indicating that individuals with *EDN3* duplication are highly inbred and homozygous. All eight Cemani and Silkie individuals possess only the *Hap*2/*Hap*4 haplotype combination (Table 2). This is in sharp contrast to the presence of the *Hap4* homozygous BKL2 and *Ha*p2/*Hap*10 heterozygous RJF9527 in RJF. The absence of segregation of *Hap*2 and *Hap*4 in Cemani and Silkie indicates that they are homozygous with respect to the single *Hap*2-*Hap*4 haplotype. In other words, *Hap*2 and *Hap*4 are no longer allelic in these breeds. This observation strongly suggests that *EDN3* was duplicated by unequal crossing over, and the two resulting loci produced permanent heterozygosity for these alleles.

**Table 2:** *EDN3* haplotypes of 36 individuals.

	Populations^c^					
Haplotypes per individual^c^ (*n*^a^)	GJF (6)	RJF (6)	Cemani (5)	Silkie (3)	Kedu (9)	Others^b^ (7)
*Hap*2	–	–	–	–	–	KAL7
*Hap*2’	–	BKL1				
*Hap*4	–	BKL2			KD1, 2, 5	KT9,KAL28
*Hap*5	Bd72, 92					
*Hap*6	–	Aceh1, 4				
*Hap*7	BOJA1					
*Hap*8	BYW5,FL9					
*Hap*10		RJF1				
*Hap*11	BYW2					
*Hap*1/*Hap*2					KDP5	
*Hap*2/*Hap*4			CM1, 6, 31, Cemani40, 43	SIB2, 17, WS3741	KD3, 16, KDH3, 8	STC13, LOM39
*Hap*2/*Hap*10		RJF9527				
*Hap*3/*Hap*4						ARS15
*Hap*4/*Hap*7					KDP7	
*Hap*4/*Hap*9						ARG19

^**a**^ *n* is the number of sampled individuals in each population:

^b^ “Others” consists of Ayam Sentul (*n* = 1), Ayam Lombok (*n* = 1), Ayam Arab (*n* = 2), Ayam Kate (*n* = 1), and Ayam Kalosi (*n* = 2):

^c^ see Table 1 for sampled individuals in each population and S3 Table for the haplotype definition.

Curiously, four Kedu (KD3, KD16, KDH3, and KDH8) and some other (STC13 and LOM39) individuals also show the same *Hap*2/*Hap*4 haplotype combination as Cemani and Silkie. As the phenotypes of KDH3 and KDH8 are quite similar to that of Cemani (S1 Fig), we speculate that these Kedu individuals are heterozygotes, each possessing one *Fm* chromosome with duplicated *EDN3* and one wild-type chromosome with a single *EDN3*. This suggests interbreeding between Cemani and Kedu, and is based on the likelihood of the allele on the wild-type chromosome being either *Hap*2 or *Hap*4, in light of their high frequencies in Indonesian chicken breeds. On the other hand, KD3 and KD16 show apparent wild-type phenotypes for comb and face color (S1 Fig), suggesting that they possess two wild-type chromosomes with distinct *Hap*2 and *Hap*4 alleles. In any case, as other individuals show different haplotypes (Table 2), the Kedu population appears to be much more heterogeneous than Cemani and Silkie with respect to haplotypes and copy number of *EDN3* genes.

**Fig 1:**
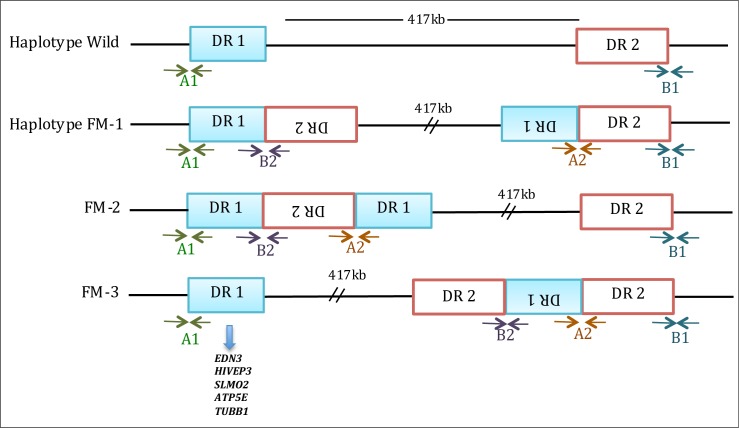
Three possible arrangements of duplicated DR1s and DR2s in the *Fm* region. Duplication of DR1 and DR2 is absent in the wild-type chromosome (top bar). A2 and B2 primer sets are designed for detecting the duplication boundaries between DR1 and DR2; A1 and B1 are for amplification control. This figure is modified from Dorshorst et al. [14]

We tested whether Cemani and the other chicken breeds have the same duplicated regions of DR1 and DR2 as Silkie does. We amplified the regions from DNA of 56 individuals using two sets of specific primers [14] (A2 and B2 in Fig 1) (Table 3). The A2 primer set is designed to amplify a region that may contain either the boundary between inverted DR1 and DR2 (1RD-DR2) or that between inverted DR2 and DR1 (2RD-DR1) in a head-to-head configuration, whereas the B2 primer set is designed to amplify a region that contains either the boundary between DR1 and inverted DR2 (DR1-2RD) or that between DR2 and inverted DR1 (DR2-1RD) in a tail-to-tail configuration. The control primer sets A1 and B1 successfully amplified target sequences in all the samples, as reported previously [14]. Amplification with A2 and B2 was consistently successful for 12 Cemani and 12 Silkie individuals and for seven Kedu samples. These findings indicate that the nucleotide sequences of 1RD-DR2/2RD-DR1 and DR1-2RD/DR2-1RD amplification products from Cemani are identical to those from Silkie (S2 Fig).

**Table 3:** PCR amplification of the duplication boundaries between DR1 and DR2 in the *Fm* region.

Samples (no. of individuals)	Positive for duplication of DR1 and DR2	Negative for duplication of DR1 and DR2
Cemani (12)	Cemani40^b^, Cemani43^b^ Cemani41, Cemani42, Cemani44-47, CM6^b^, CM11, CM31^b^, Cemani	–
Silkie (12)	SIB2^b^, SIB6, SIB7, SIB11, SIB14, SIB15, SIB16, SIB17^b^, KPS16, KPS17, KPS18, KPS30	–
Kedu (24)	KDH3^b^, KDH8^b^, KD16^b^, KD17, KD19, KD21, KD22	KD1^d^, KD2^d^, KD3^b^, KD4, KD5^d^, KD7-15, KDP1, KDP5^a^, KDP7^e^,
Other chicken breeds (4)	–	STC13^b^, LOM39^b^, BMC610, BMC613,
Jungle fowls (4)	–	BKL1^c^, BKL2^d^, Bd72^f^, Bd92^f^

^a-f^ *EDN3* haplotypes of 21 individuals are the same as those indicated in Table 2;

^a^ *Hap*1/*Hap*2,

^b^ *Hap*2/*Hap*4,

^c^ *Hap*2’,

^d^ *Hap*4,

^e^ *Hap*4/*Hap*7,

^f^ *Hap*5.

In addition, 35 individuals in Table 1 with unknown *EDN3* haplotypes are examined for the duplication.

The duplication boundary is identified in all Cemani and Silkie individuals, and also in some Kedu individuals.

We confirmed that the *Hap*2/*Hap*4 combination in STC13 and LOM39 does not result from duplicated DR1, but stems from the segregation of the *Hap*2 and *Hap*4 alleles. However, the results of the A2 and B2 amplifications for the 24 Kedu individuals were somewhat puzzling. Amplification was successful for KDH3, KDH8, and KD16, but not for KD3, despite the fact that all four carry the *Hap*2/*Hap*4 combination. These observations for KDH3, KDH8, and KD3 agree with the aforementioned speculation that KDH3 and KDH8 have at least one *Fm* chromosome, while KD3 has two wild-type chromosomes. However, the result for KD16 is unexpected and suggests that, despite its wild-type phenotype, KD16 might possess at least one *Fm* chromosome. This speculation is supported by the presence of noticeable black pigment on the comb of KD16 (S1 Fig). This may also be true for KD17, KD19, KD21, and KD22 samples which exhibited successful A2 and B2 amplification (Table 3 and S1 Fig). This observation corroborates the high heterogeneity of *Fm* in the Kedu population. Although it is conceivable that the heterogeneity is related to Cemani breeding in the same geographic area of Central Java, further investigation of the genotype-phenotype relationship is required to draw any definitive conclusion. The high heterogeneity in the Kedu Fm phenotype also suggests that the *Dermal Melanin* inhibitor (*ID*) locus, on chromosome Z [44], is worth further investigation.

**Fig 2:**
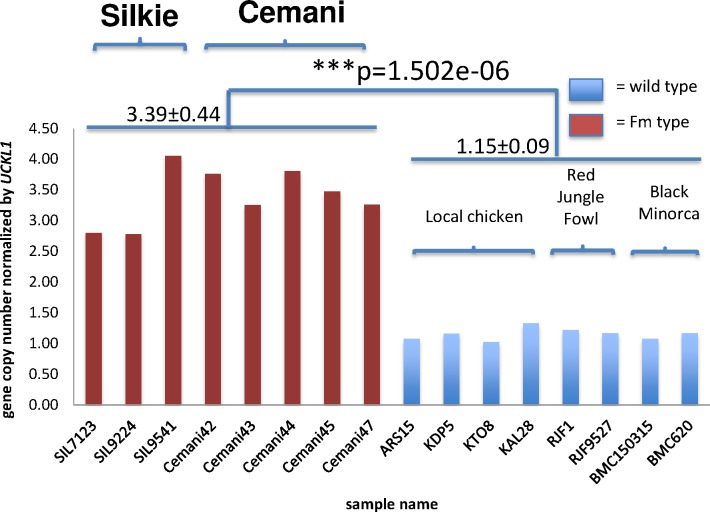
*EDN3* CNV. Copy numbers of *EDN3* were normalized to those of *UCKL1* in qPCR. Red bars represent the copy numbers of three Silkie and five Cemani individuals, and blue bars represent those of eight wild-type chickens. The average copy number in *Fm*-phenotype chickens is 3.39 ± 0.44 and that in wild types is 1.15 ± 0.09 (*P* = 1.50 × 10^−6^).

**Fig 3:**
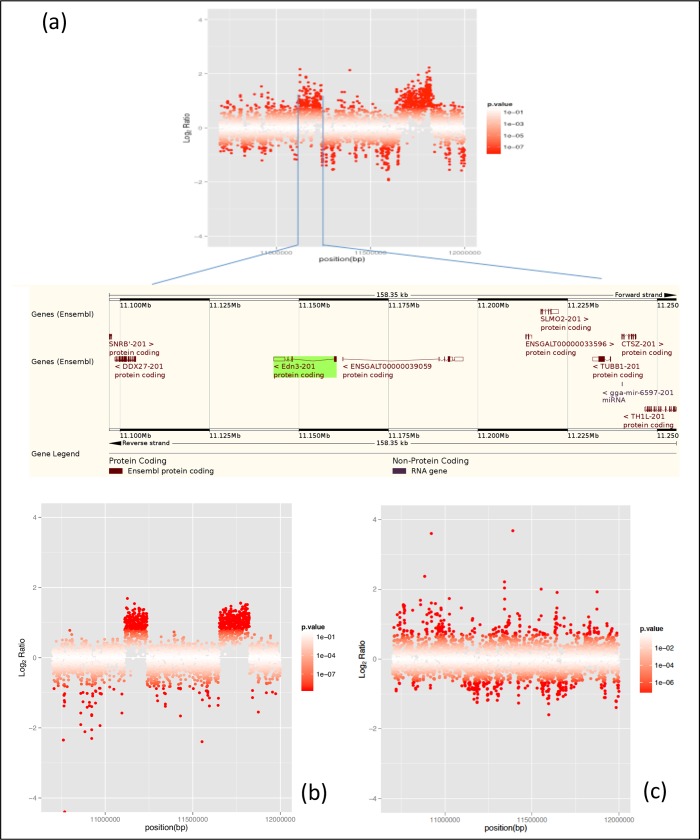
*Log*_2_ ratio for CNV in chicken chromosome 20 between nt positions 10,700,000 and 12,000,000. (a) Comparison of read mapping between Cemani and Taiwanese L2. A blow-up of the DR1 region containing *EDN3* is shown below the map. Comparison of read mapping between combinations of (b) Silkie and Taiwanese L2 and (c) Cemani and Silkie.

Second, we studied CNV in *EDN3* using qPCR. We measured the absolute concentrations of amplified *EDN3* and *UCKL1* amplicons in reaction mixtures of each sample and normalized the copy number of *EDN3* based on the single-copy gene of *UCKL1*. Cemani and Silkie have twice to four times larger copy numbers than non-Fm chickens (Fig 2). Although the exact number of *EDN3* copies in Cemani and Silkie genome is difficult to estimate by the qPCR alone, the Fm phenotype surely shows excessive *EDN3* copies [12]. In addition, we carried out WGS for a single Cemani individual (Cemani 41). Using CNV-Seq [31], we confirmed that approximately twice as many reads were mapped onto the DR1 and DR2 in Cemani as compared to Taiwanese native chicken L2 with non-Fm phenotype (Fig 3A) and a similar result was obtained in Silkie with respect to L2 (Fig 3B) [23]. However, when the Cemani genome was compared with the Silkie genome, neither DR1 nor DR2 showed any excess of reads (Fig 3C). Together, these results consistently indicate that the DR1 and DR2 arrangement in Cemani is identical to that in Silkie and strongly support a single common origin of the Fm phenotype in Cemani and Silkie.

### Haplotype diversity and duplication of *EDN3*

To study the origin of *Hap*2 and *Hap*4 at duplicated *EDN3* loci, we examined the sequence differences among the 12 haplotypes or alleles in more detail. The haplotype sequences of the 36 individuals (Table 2) contain 35 variable sites consisting of one 1-bp deletion, two 3-bp deletions, and 28 point mutations (S3 Table). Of these haplotypes, five are singletons in the sample, whereas *Hap*2 and *Hap*4 are represented in 17 and 23 individuals, respectively. *Hap*2’ is one of the singletons and differs from *Hap*2 by a single point mutation at position 784. As it occurs in RJF, it has likely been derived from *Hap*2 in the RJF population. Likewise, *Hap*9 differs from *Hap*4 by a single 3-bp deletion and descends in indigenous Ayam Arab ARG19. More importantly, *Hap*10 was found only in RJF and differs from *Hap*2 and *Hap*4 by one and two point mutations, respectively. Therefore, *Hap*10 likely is a common ancestor of *Hap*2 and *Hap*4. Thus, the allelic divergence among *Hap*2, *Hap*4, and *Hap*10 must have occurred in the RJF population, which still harbors all these alleles.

**Fig 4:**
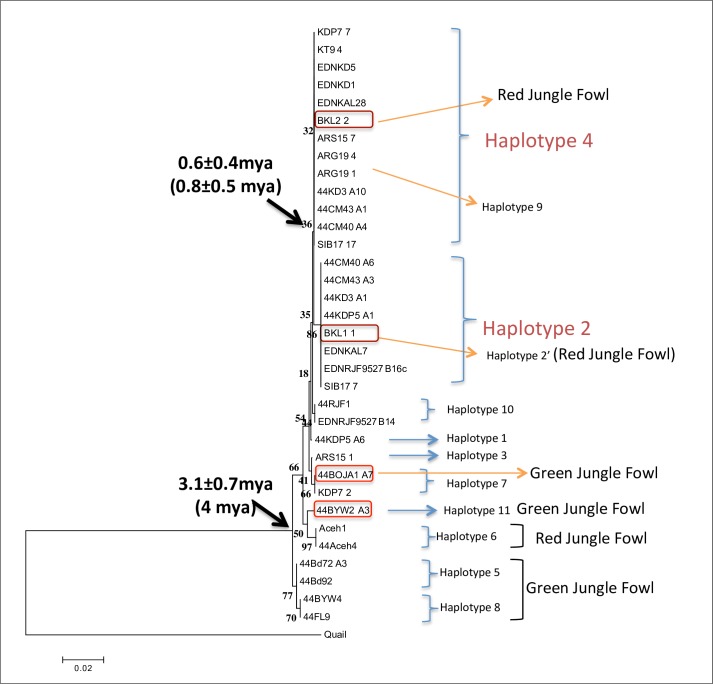
NJ tree of *EDN3* haplotypes rooted by the quail sequence (accession number NC_029535). The tree was constructed with 1000 bootstrap resampling with an option of complete deletion of gaps/missing nucleotides [33]. The nucleotide divergence was measured by using the number of nucleotide differences per site, without multiple-hit correction.

To examine the phylogenetic relationship among the 12 haplotypes, we constructed an NJ tree [33] rooted by the orthologous quail sequence and statistically evaluated with 1000 bootstraps based on all nucleotide substitutions in 34 1-kb fragments derived from 29 individuals (Fig 4). Although the tree showed several intermingling patterns of ancestral allelic lineages leading to RJF and domesticated chickens owing to incomplete lineage sorting, it did support that *Hap*10 is a recent common ancestor of *Hap*2 and *Hap*4. Next, we estimated the divergence time between *Hap*2 and *Hap*4 based on two calibrated substitution rates. One is based on the published substitution rate in introns [8,45]. When the rate of (1.7–1.8) × 10^−9^ per site per year is applied to the average per-site nucleotide differences between *Hap*2 and *Hap*4 (0.0020 ± 0.0014), the divergence time of 0.6 ± 0.4 million years is obtained. Alternatively, we can directly calibrate the substitution rate in the present *EDN3* sequences using the divergence time of RJF/domesticated chickens from GJF. This divergence time can be inferred from such a geological event as the emergence of Java island 3–4 million years ago [46]. Further evidence from fossil records regarding the 4–5 million year-old ancestor of *Gallus* (*Gallus bravardi*) consistently suggests that GJF originated around 4 million years ago [47,48]. In this calibration, however, it has to be noted that four distinct haplotypes (*Hap*5, 7, 8, and 11) exist in GJF, of which *Hap*11 clustered together with *Hap*6 in RJFs (Aceh1 and Aceh4), and *Hap*7 is the same haplotype as that in the domesticated KDP7. Provided that GJF is indigenous to Java and the Lesser Sunda Islands, these *Hap*7 and *Hap*11 in GJF raise the possibility of recent introgression from RJF and/or domesticated chickens [8]. Therefore, we excluded *Hap*7 and *Hap*11 when calculating the average nucleotide difference per site (0.0107 ± 0.0025) between GJF and other chickens, which resulted in the substitution rate of 1.3 × 10^−9^ per site per year. This rate is a little slower than the previous one and yields a somewhat earlier estimate of the divergence time (0.8 ± 0.5 million years) between *Hap*2 and *Hap*4. In either case, a rough estimate of divergence time (0.6–0.8 myr) implies that these *EDN3* alleles in fact originated in the ancestral RJF population. At some point after this allelic divergence, the *EDN3* locus was duplicated, and the Fm phenotype appeared. We will discuss a lower limit of this allelic divergence that can be set by the divergence time between the Cemani and Silkie breeds, together with a re-evaluation of the above estimates with large standard errors.

### High- homozygosity tracts (HHTs) in Cemani and Silkie

**Fig 5:**
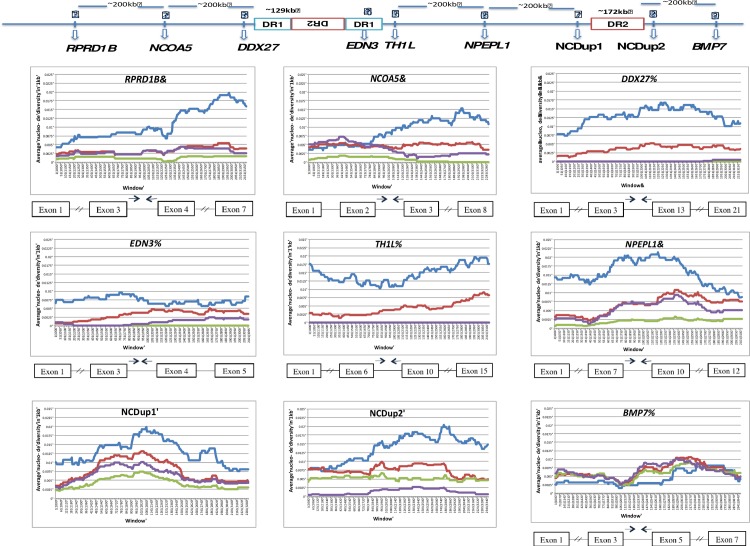
The nucleotide diversity (*π*) in nine regions surrounding *END3* in chicken breeds. The ***π*** values in Cemani (green), Silkie (purple), other domesticated breeds (red), and GJF (blue) are shown under a schematic diagram of their locations together with duplicated DR1 and DR2 regions on chromosome 20. Each *π* is measured in a 1 kb window with an overlapping sliding size of 100 bp. All regions except 7 and 8 are located within genes whose exon and intron structures are indicated below diversity plots.

To detect any genomic signature of artificial selection on the Fm phenotype, we investigated the pattern and degree of DNA polymorphism in DR1 and its surrounding genomic regions. Using 9 Cemani, 10 Silkie, 11 other domesticated chickens including a single RJF, and two GJFs, we first examined nine regions of about 3 kb long and separated by ~200-kb intervals. As a whole, they span a 1.4-Mb genomic region that includes the 254-kb duplicated DR1s, 342-kb duplicated DR2s, and 413-kb spacer (Fig 5 and S3 Fig). Table 4 shows summary statistics of the genetic variability in these nine regions (see S4 Fig for NJ trees). First, the number of haplotypes (*H*_*k*_) in a sample of *k* chromosomes is generally much smaller than the number of segregating sites (*S*_*k*_) within the same region. Each region is thus in fairly strong linkage disequilibrium and is consistent with relatively large values of |*D*′| (not shown) or the squared correlation coefficient (*r*^2^): the absolute value of *r* is greater than 0.56 in all regions of all four populations. Second, the pattern and level of DNA polymorphism in Cemani and Silkie greatly differ from those in “Others” and GJF. We note that region 3 is located upstream of DR1, region 4 is within DR1, and regions 5–7 are within the spacer, while regions 1, 2, 8, and 9 are further away from the *Fm* region. Compared with regions 1, 2, and 6–9, regions 3–5 in Cemani and Silkie show a remarkable reduction in *H*_*k*_, *S*_*k*_, Watterson’s *θ*_*w*_ and nucleotide diversity *π* [49–51]. For instance, in regions 1, 2, and 6–9, the average number of segregating sites per kb is about 12 in Cemani and Silkie. The expected number in each 3-kb region is thus about 30; however the actual number observed in regions 3 and 5 is 0 in Cemani and at most 2 in Silkie. We regarded this extremely low level of genetic variability as evidence for selective sweep via artificial selection for *Fm*. To verify this, we carried out the HKA test for Cemani and Silkie [34] using the divergence data in comparison with GJFs. The test indicated a significantly lower level of polymorphism in regions 3–5 than in any other region (S5 Fig). Additionally, we applied the STRUCTURE analysis region by region [35]. Although Cemani and Silkie individuals are generally assigned to different multiple genetic components in regions 1, 2, and 6–9, they are assigned to a single common component in regions 3–5 (*DDX27*, *EDN3*, and *TH1L*) (S6 Fig).

**Table 4:** DNA polymorphism in nine regions in three populations of chickens and GJF (see also Fig 5).

		Populations			
Region (*L* bp) and gene in it	Statistics^a^	Cemani	Silkie	Others	GJF
	*k*^b^	18	20	22	4
R1 (3138)	*H*_k_ (*E*(*H*_k_))	3 (7.0)	8 (10.5)	12 (12.1)	4 (3.8)
*RPR1D*	*S*_k_ (*θ*_w_)	21 (0.19)	25 (0.23)	40 (0.35)	56 (0.97)
	*π* = *θ* (± SE)	0.12 (0.03)	0.26 (0.05)	0.33 (0.06)	1.06 (0.14)
	*D* (*r*^2^)	0.064 (0.59)	0.060 (0.44)	0.044 (0.32)	0.12 (0.49)
R2 (3299)	*H*_k_ (*E*(*H*_k_))	2 (5.1)	8 (11.9)	12 (13.7)	3 (3.8)
*NCOA5*	*S*_k_ (*θ*_w_)	10 (0.09)	33 (0.28)	57 (0.47)	44 (0.73)
	*π* = *θ* (± SE)	0.06 (0.02)	0.35 (0.07)	0.44 (0.06)	0.82 (0.12)
	*D* (*r*^2^)	0.099 (1.0)	0.054 (0.42)	0.046 (0.35)	0.14 (0.57)
R3 (3153)	*H*_k_ (*E*(*H*_k_))	1 (1)	2 (2.8)	12 (12.2)	3 (3.8)
*DDX27*	*S*_k_ (*θ*_w_)	0 (0)	2 (0.02)	41 (0.36)	54 (0.93)
	*π* = *θ* (± SE)	0	0.02 (0.01)	0.33 (0.07)	1.14 (0.16)
	*D* (*r*^2^)	N.A.	0.090 (1.0)	0.043 (0.31)	0.24 (0.96)
R4 (3093)	*H*_k_ (*E*(*H*_k_))	2 (3.4)	3 (7.6)	10 (11.3)	3 (3.8)
*EDN3*	*S*_k_ (*θ*_w_)	2 (0.02)	13 (0.12)	40 (0.35)	37 (0.65)
	*π* = *θ* (± SE)	0.03 (0.02)	0.13 (0.03)	0.28 (0.05)	0.72 (0.13)
	*D* (*r*^2^)	0.099 (1.0)	0.083 (0.77)	0.051 (0.49)	0.13 (0.52)
R5 (3110)	*H*_k_ (*E*(*H*_k_))	1 (1)	1 (1)	16 (14.2)	4 (3.9)
*TH1L*	*S*_k_ (*θ*_w_)	0 (0)	0 (0)	57 (0.50)	82 (1.44)
	*π* = *θ* (± SE)	0	0	0.52 (0.09)	1.55 (0.18)
	*D* (*r*^2^)	N.A.	N.A.	0.048 (0.39)	0.12 (0.48)
R6 (3086)	*H*_k_ (*E*(*H*_k_))	4 (8.4)	9 (12.9)	14 (14.6)	4 (3.9)
*NPEPL1*	*S*_k_ (*θ*_w_)	34 (0.32)	35 (0.32)	58 (0.52)	70 (1.24)
	*π* = *θ* (± SE)	0.18 (0.03)	0.47 (0.09)	0.58 (0.08)	1.42 (0.18)
	*D* (*r*^2^)	0.078 (0.70)	0.056 (0.45)	0.045 (0.33)	0.17 (0.66)
R7 (3286)	*H*_k_ (*E*(*H*_k_))	8 (11.5)	10 (13.7)	13 (15.4)	3 (3.9)
*NCdup1*	*S*_k_ (*θ*_w_)	55 (0.49)	55 (0.47)	65 (0.54)	64 (1.06)
	*π* = *θ* (± SE)	0.39 (0.06)	0.54 (0.08)	0.66 (0.09)	1.18 (0.15)
	*D* (*r*^2^)	0.074 (0.64)	0.057 (0.48)	0.051 (0.44)	0.13 (0.52)
R8 (3342)	*H*_k_ (*E*(*H*_k_))	9 (12.3)	4 (7.3)	20 (15.8)	4 (3.9)
*NCdup2*	*S*_k_ (*θ*_w_)	38 (0.33)	21 (0.18)	76 (0.62)	71 (1.16)
	*π* = *θ* (± SE)	0.47 (0.09)	0.11 (0.02)	0.70 (0.09)	1.28 (0.16)
	*D* (*r*^2^)	0.071 (0.59)	0.073 (0.73)	0.045 (0.33)	0.092 (0.37)
R9 (3960)	*H*_k_ (*E*(*H*_k_))	12 (13.4)	14 (14.8)	14 (15.7)	3 (3.7)
*BMP7*	*S*_k_ (*θ*_w_)	65 (0.48)	76 (0.54)	81 (0.56)	27 (0.37)
	*π* = *θ* (± SE)	0.56 (0.08)	0.61 (0.08)	0.58 (0.06)	0.41 (0.07)
	*D* (*r*^2^)	0.060 (0.44)	0.052 (0.38)	0.044 (0.33)	0.13 (0.53)

^a^ *H*_*k*_ is the observed number of haplotypes in a sample of *k* chromosomes. *E*(*H*_*k*_) is based on the formula for the expected number of neutral alleles with per-locus mutation rate *θL*, where *θ* is given by the observed *π* value and *L* is the number of nucleotides per region. *S*_*k*_ is the observed number of segregating sites within a region. *θ*_*w*_ is Watterson’s *θ* and *π* = *θ* is nucleotide diversity, both were multiplied by 100. *D* is the mean value of linkage disequilibrium across all pairs of polymorphic sites within a region, and *r* is the corresponding correlation coefficient given by $r=\frac{D}{\sqrt{p_{A}q_{A}p_{B}q_{B}}}$, where the denominator is proportional to heterozygosity at both sites *A* and *B*.

Among regions 3–5, region 4 within DR1 shows an exceptionally high level of genetic variability; however, this is deceptive and results from the inevitable mixture of duplicated sequences. The homologous sequences within duplicated DR1 cannot be amplified separately by the present PCR method; genetic variability in region 4 within DR1 is simply owing to a mixture of the two paralogous sequences. Despite this caveat, the level of genetic variability in region 4 is somewhat higher in Silkie than in Cemani. This is largely due to the inclusion of three sequences of Indonesian Silkie (KPS16, 17, and 30) and is also visible in the STRUCTURE analysis (S6 Fig). The nucleotide sites at positions, 1828, 2015, 2049, 2230, 2279, 2630, 2637, 2664, 2837, and 3005 in region 4 (ranging from 11,157,992 to 11,158,169 in the reference genome) are variable with respect to two distinct haplotypes. One haplotype is identical to that in Silkie and the other is identical to that in indigenous Indonesian chicken breed KAL28 (see NJ tree of region 4 in S4 Fig), suggesting frequent occurrence of interbreeding between Indonesian Silkie and other indigenous domesticated chickens.

**Fig 6:**
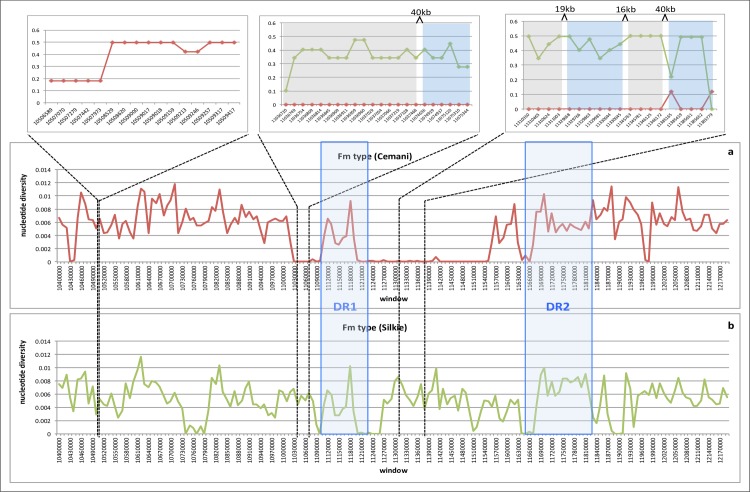
Nucleotide diversity (*π*) based on NGS genotype data for one Cemani (red) and one Silkie (green) individual. Diversity is measured in 10-kb non-overlapping windows. The left-shaded region represents DR1, in which nearly the same patterns and degrees of polymorphism are exhibited by Cemani and Silkie. This supports that DR1 was duplicated prior to their diversification and has since been frozen from recombination in both breeds. The same trend is observed in the right-shaded DR2; however, the ancestral haplotype is obscured by recombination. The three upper panels show the proportion of per-SNP heterozygotes at the population level for Cemani (red) and Silkie (green). Observations are made in 9 windows, each 1 kb long. The 9 windows are grouped into sets of 3, 2, and 4 windows. The three windows at the left are consecutive and contain 16 SNPs in total. The two windows in the middle and four at the right are separated by 16–40 kb and contain 22 and 19 SNPs, respectively.

We aimed to determine whether the regions with reduced genetic variability or the HHTs are identical for Cemani and Silkie. For practical purposes, we operationally defined an HHT as a consecutive genomic region over 10 kb with *π* < 10^−4^ or < 2% of the normal level *π*_0_ = 0.005 (Table 4). We determined the WGS of one Cemani individual and compared it with those of Silkie and Taiwanese L2. Cemani and Silkie exhibit a similar extent of reduction in DR1 and its surrounding regions, but the HHT length differed greatly between these two breeds (Fig 6). The Cemani HHT is long and extends toward regions 2 and 7, whereas the Silkie HHT is relatively short and limited from a little beyond regions 3 to 5. The left and right HHT lengths are, respectively, 118 and 387 kb in Cemani and 52 and 101 kb in Silkie, respectively. However, it is highly probable that such a tract can differ from individual to individual even within a breed. Therefore, using the same samples of 19 Cemani and Silkie in Table 4, we further examined the genetic variability surrounding the boundaries in nine windows, each about 1 kb each in length (Fig 6). The windows are localized into three clusters; the left HHT in Cemani still extends beyond the central cluster, while that of Silkie already ends there. Likewise, the right HHT in Cemani terminates just within the right cluster, whereas that in Silkie ends well within in this cluster. We measured the left and right HHT from the 5’ and 3’ ends, respectively, of *EDN3* (11,144,657–11,161,475) and estimated the tract lengths. The minimum left and right HHT lengths are 118 and 224 kb, respectively, in Cemani and 52 and 101 kb, respectively, in Silkie (Fig 6).

## Discussion

### Genomic configuration of the *Fm* region

Dorshorst et al. [14] proposed three possible rearrangements (FM1–FM3) of duplicated DR1s and DR2s in the *Fm* region (Fig 1). Although all these rearrangements possess the same boundaries of 1RD-DR2/2RD-DR1 and DR1-2RD/DR2-1RD, one major difference exists with respect to the relative position of the 413-kb spacer. In models FM1 and FM3, duplicated DR1s sandwich the spacer. If either FM1 or FM3 is valid, the HHT is expected to cover the entire spacer as the two *EDN3* loci in DR1s are simultaneous targets of artificial selection. In contrast, in model FM2, the spacer is located outside the duplicated DR1s, and can therefore recombine with wild-type chromosomes without disrupting the Fm phenotype. In this case, the spacer region is expected to be polymorphic because of recombination. Our data (Figs 5 and 6 and Table 4) clearly showed that the patterns and degrees of polymorphism exhibited by Cemani and Silkie are consistent with FM2, but inconsistent with FM1 and FM3.

### DR1 duplication and emergence of the Fm phenotype

We estimated an upper limit of *EDN3* duplication time of 0.6 ± 0.4 ∼ 0.8 ± 0.5 million years based on the allelic divergence between *Hap*2 and *Hap*4. Although the standard error is large because of the usage of the short sequences, *Hap*2 and *Hap*4 seem to diverge from each other much earlier than is documented in any known archeological record of domesticated chickens. As mentioned earlier, *EDN3* duplication and the Fm phenotype emerged in the ancestral RJF population of chickens; therefore, this phenotypic variation was highly likely to be selected once domestication began in Asia. Xiang et al. [52] dated ancient mtDNA sequences from the earliest archaeological chicken bones in China back to 10,000 years ago.

The analysis of the Cemani and Silkie genome sequences revealed that the 71.4-kb region spanning nt 11,183,600 to 11,255,000 is located within the joint set of the right HHTs in both breeds (Fig 6 and S7 Fig). In this region, recombination has been apparently prohibited by artificial selection on *EDN3* and therefore, the two breeds are most closely related to each other in terms of nucleotide substitutions (S7 Fig). Because of the paralogy between DR1s, 50 variable sites in Cemani and 51 in Silkie are observed within a stretch of an approximately 55 kb of 71.4-kb region. It is important to note that a great majority (49) of these variable sites are shared between the two breeds, implying that they accumulated in their common ancestor (S4 Table). As the per-site differences amount to approximately (9.2 ± 1.3) × 10^−4^, we can estimate the sequence divergence time between the duplicated DR1s as 0.26 ± 0.04 ∼ 0.35 ± 0.05 million years ago. These are more recent, but more reliable than the previous estimates for the upper limit of DR1/*EDN3* duplication time. In either case, we conclude that the Fm phenotype caused by duplicated DR1/*EDN3* originated in RJF long before the domestication process began in China.

**Fig 7:**
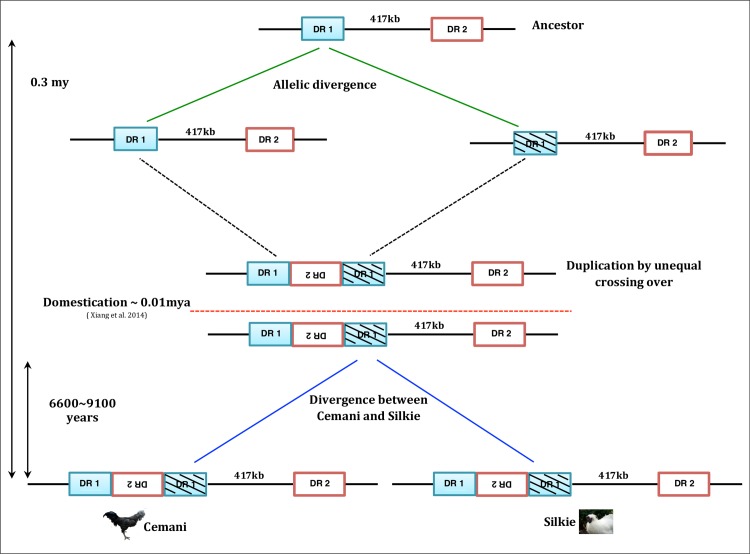
Evolutionary history of *EDN3* genes in Cemani and Silkie. The green lines represent the allelic divergence of *Hap*2 and *Hap*4 at *EDN3* in the ancestral RJF population. The blue lines represent the ancestral lineages in the 71.4-kb region (shadow), from which the divergence time between Cemani and Silkie was estimated. The DR1 duplication is placed somewhere between 10,000 and 300,000 years ago. The horizontal red line corresponds to the beginning of the domestication process in China [52].

Additionally, we are interested in the divergence time between Cemani and Silkie, to use it as a lower limit for the DR1 duplication time. For this purpose, we used breed-specific substitutions. Provided that recombination is rare or absent within the 71.4-kb region, we treated such substitutions as derived variants and proportional to the divergence time between Cemani and Silkie breeds. In the entire region of 2 × 55 + 16 = 126 kb, there are one Cemani-specific and two Silkie-specific nucleotide substitutions (S4 Table). The mean per-site sequence differences are therefore given by $d=\frac{3}{126000}=2.4\times 10^{-5}$ for a pair of Cemani and Silkie genomes. Using the calibrated nucleotide substitution rate of (1.3 ∼ 1.8) × 10^−9^, we obtain the divergence time of 6600 ∼ 9100 years and regard it as an upper limit of the divergence time between the Cemani and Silkie breeds. Because this also gives a lower limit of the DR1/*EDN3* duplication time, the estimate suggests that the Fm phenotype emerged by this time (Fig 7).

### Ayam Cemani and Ayam Kedu in Indonesia

The extent of nucleotide diversity in Cemani is almost the same as that in other domesticated chickens and jungle fowl, except near the *EDN3* locus (regions 3, 4, and 5), despite the fact that Cemani is a local breed of Kedu, in Indonesia. This is in sharp contrast to Silkie which we could be sampled in Indonesia, Japan, and the USA in the present study. There are two possible explanations for this high genetic variability in Cemani: a relatively large founding population, and frequent genetic exchanges with other domesticated chickens or jungle fowls. The presence of KDH3 and KDH8, which are heterozygous for the *Fm* and wild-type chromosomes, supports the latter hypothesis of interbreeding of Cemani with other domesticated chickens. In this case, there must have been intense selection to maintain the Fm phenotype. However, the effect of intense selection can be limited in genomic regions closely linked to a target site. Further study of Ayam Kedu with abundant *Fm* variation will provide useful information on their breeding schemes and the history of the *Fm* phenotype in Indonesia.

### History and strength of artificial selection

We used information on HHTs in two simple ways [53] without any sophistication for inference [54–56]. One is to infer the allele age or the history of artificial selection based on the idea underlying the inference of adaptively introgressed tracts [38,39], and the other is to infer the strength of artificial selection, as has been done for maize domestication [1]. Both estimates depend heavily on the recombination rate *r* per bp per generation. The recombination rate is known to vary considerably among as well as within chromosomes [7]. The rate is 3.7 cM/Mb if averaged over the entire chicken genome, whereas it is approximately 3.0–5.0 cM/Mb for chromosome 20. We assume here *r* = 3.0 cM/Mb = 3.0 × 10^−8^ per bp per generation. When considering effect of inbreeding in the domestication process, *r* is replaced with the effective recombination rate r^*^ = *r*(1 − *f*) in which *f* is the inbreeding coefficient [39].

As mentioned previously, we measured the minimum lengths of the left and right HHTs in sequences of Cemani or Silkie individuals. Based on both the population and NGS data, the left and right HHT lengths are 118 kb and 224 kb, respectively, in Cemani, and 52 kb and 101 kb, respectively, in Silkie. Using formula (1) [37, 38], with an observed mean tract length $\hat{x}$, we calculated the number of generations elapsed under artificial selection (see Materials and Methods). In the case of Cemani, $\hat{x}=\frac{118+224}{2}=171$ kb so that *t* ≈ 200/(1 − *f*), whereas in the case of Silkie, $\hat{x}=\frac{52+101}{2}=77$ kb so that *t* ≈ 440/(1 − *f*). It thus appears that the history of Cemani is approximately half of that of Silkie. In the absence of inbreeding, the tract erodes quickly, but even intense inbreeding such as full-sib mating, with *f* = 1/4, can increase the time only by 30%. Furthermore, if we define the generation time of chickens and fowls based on the mean age (*m*) of hens at a given time [39], we can convert the above *t* generations into *m* × *t* years. If chickens can lay eggs for 7 years (with the age at first reproduction being 1 year and the mean longevity being approximately 15 years), it might be reasonable to assume *m* = 3–4. Therefore, it appears that Cemani and Silkie have been bred for roughly (600 ∼ 800)/(1 − *f*) years and (1300 ∼ 1700)/(1 − *f*) years, respectively. Thus, the history of Indonesian Ayam Cemani appears to be rather short, whereas the relatively long history of Silkie is consistent with the relatively short HHT length in its *Fm* region.

Second, we used formula (2) for the expected nucleotide diversity *π* at linked neutral sites under recent selective sweep with selection coefficient *s* (see Materials and Methods). In the virtual absence of variation, we can have such a rough relationship as *s* = 200r^*^x. With *r* = 3 × 10^−8^ [7,57] and *x* ≥ 100 kb, we have *s* ≥ 0.6(1 − *f*). This is inevitably a crude estimate but it indeed suggests intense artificial selection in both the Cemani and Silkie breeds.

As a final caveat, it may be asked why the tract boundaries in the NGS data separate HHT very sharply from the neutral level (Fig 5). The two *Fm* chromosomes in an individual must be flanked by wild-type chromosomes and likely have different recombination breakpoints. However, the two *Fm* chromosomes have virtually identical DNA sequences in the focal site and nearby linked sites, but are different from wild-type chromosomes, which might also differ from each other. Therefore, we can identify only sharp HHT boundaries in a single diploid *Fm* individual. However, as these abrupt boundaries can differ among individuals, the tract boundaries might become more gradual for large samples or at the population level. This would explain intermediate values of *π* observed in regions 1, 2 and 6 as well as in the right HHT in a sample of nine Cemani individuals (Table 4, Fig 6).

## Conclusions

We showed that fibromelanosis (Fm) in Indonesian Ayam Cemani and Chinese Silkie resulted from the same genetic change involving *EDN3* duplication on chromosome 20. This genomic change of a single origin arose spontaneously in the ancestral population of RJF in Asia, probably well before the first domestication of chickens. Strong artificial selection for the Fm phenotype is evident in the genetic variability near the target site of duplicated *EDN3*, although the pattern and level of variability differ sensitively between these two breeds, which have undergone different domestication processes.

## Supporting information

S1 Fig Characteristic morphological traits of several Indonesian chickens and Chinese Silkie. (a) Female Cemani, (b)—(d) female Kedu, (e)—(i) male Kedu, (j) male white Silkie and (k) male black Silkie.
(TIF)

S2 Fig Sequence information for duplication boundaries generated by the A2 and B2 primer sets. The A2 and B2 sequences of Cemani (CM6_A2 and CM6_B2) are identical to those of Silkie (SIB17_A2 and SIB17_B2). The boundary was determined by comparison between A1 (CM31_A1) and A2 (upper panel), and between B1 (CM6_B1) and B2 (lower panel).
(TIF)

S3 Fig Expected heterozygosity at individual SNP sites in the nine regions in chicken breeds. (a) Domesticated chickens, RJF, and GJF, (b) Ayam Cemani, (c) Silkie chicken.
(TIF)

S4 Fig NJ trees for the nine regions in domesticated chicken breeds, and RJF. The phylogenetic relationship differs greatly from region to region. Two GJF haplotype sequences were used as outgroups.
(TIF)

S5 Fig **Results of the HKA test in each of the nine regions of Cemani (a), Silkie (b), and other domesticated chickens (c).** The significant reduction in DNA polymorphism is found in Cemani and Silkie only for *DDX27* in region 3, *EDN3* in region 4, and *TH1L* in region 5 are compared.
(TIF)

S6 Fig STRUCTURE analysis of each of the nine regions of GJF, RJF, Cemani, Silkie and other domesticated chickens. For regions 3–5, Cemani and Silkie exhibit nearly identical genetic components, whereas in other regions, there are no noticeable structural differences among chicken breeds and RJF.
(TIF)

S7 Fig Nucleotide diversity between Cemani and Silkie based on NGS data. Bars with R1–R9 indicate the positions of the nine regions. Green square parentheses indicate the position of *EDN3*, and a red bar indicates the 71.4-kb region with low divergence between the two breeds.
(TIF)

S1 Table Sequences of primers used in this study. (PDF)

S2 Table Reaction mixtures and PCR conditions used in this study. (PDF)

S3 Table Segregating sites in *EDN3* haplotypes. (PDF)

S4 Table Variable sites in the 71.4-kb region in Cemani, Silkie and Taiwanese L2 as compared to the reference genome. The region ranges from nt 11,183,600 to 11,255,000 and includes part of DR1. Insertions and deletions are excluded. The colored columns indicate the Silkie (green)- or Cemani (red)-specific mutations.
(PDF)
