# Supplementary figures and images for: The origin and evolution of fibromelanosis in domesticated chickens: Genomic comparison of Indonesian Cemani and Chinese Silkie breeds
Source: PLoS One. 2017 Apr 5;12(4):e0173147. doi: 10.1371/journal.pone.0173147 (PMC5381777; doi:10.1371/journal.pone.0173147)

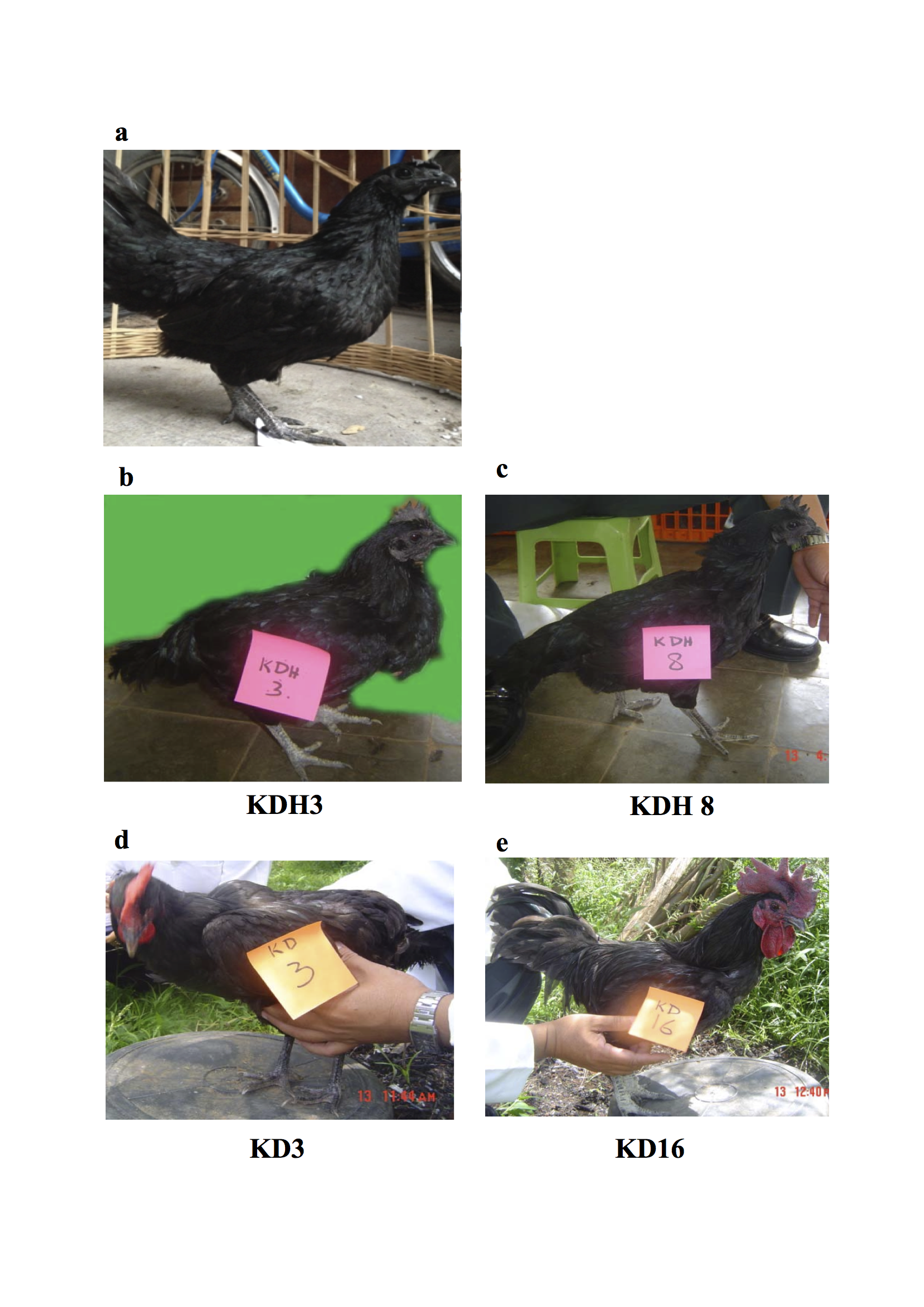

Supplement: S1 Fig — (a) Female Cemani, (b)—(d) female Kedu, (e)—(i) male Kedu, (j) male white Silkie and (k) male black Silkie. (TIF) [file pone.0173147.s001.tif]

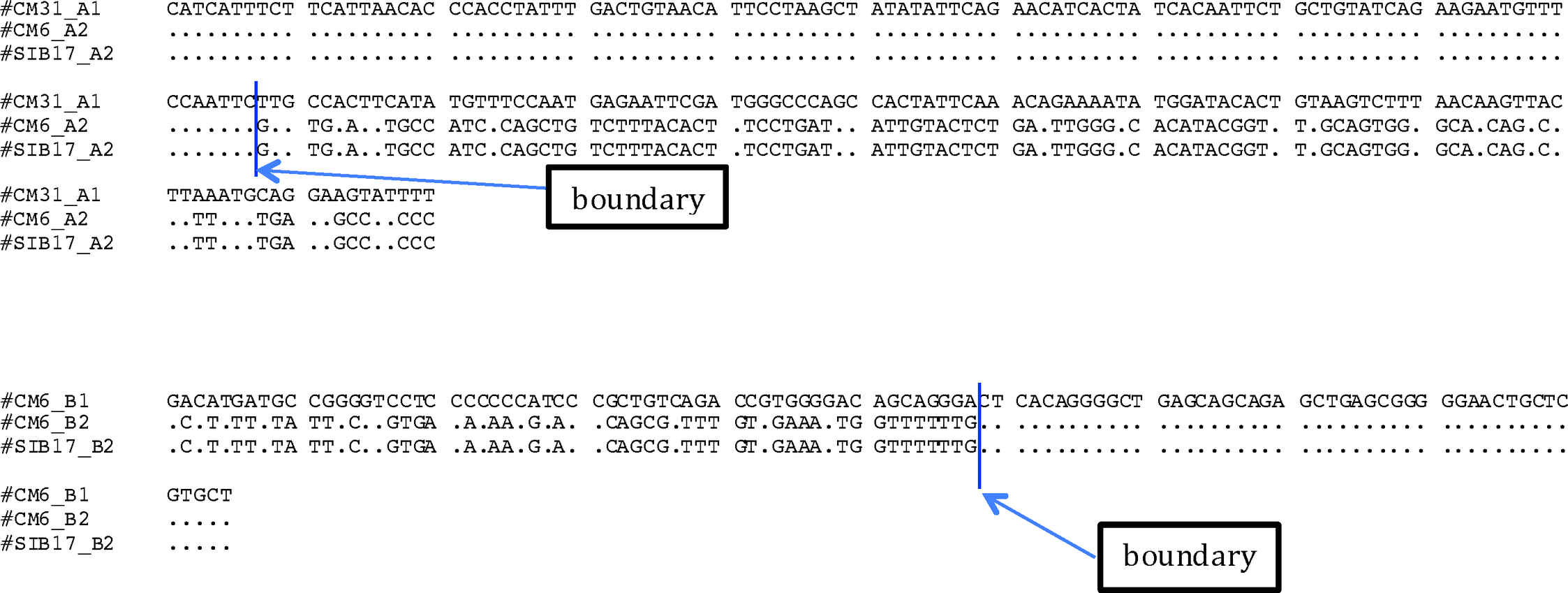

Supplement: S2 Fig — The A2 and B2 sequences of Cemani (CM6_A2 and CM6_B2) are identical to those of Silkie (SIB17_A2 and SIB17_B2). The boundary was determined by comparison between A1 (CM31_A1) and A2 (upper panel), and between B1 (CM6_B1) and B2 (lower panel). (TIF) [file pone.0173147.s002.tif]

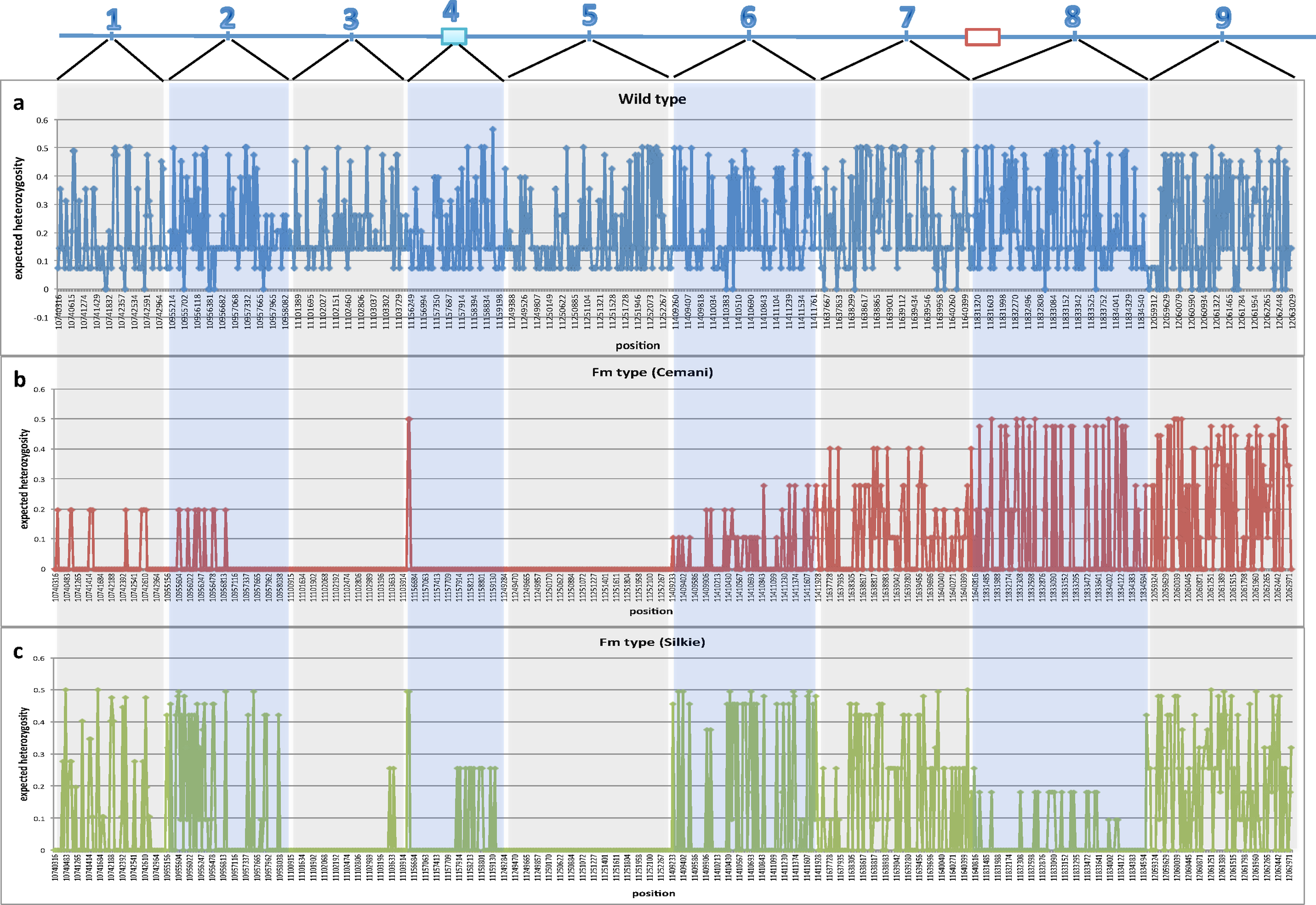

Supplement: S3 Fig — (a) Domesticated chickens, RJF, and GJF, (b) Ayam Cemani, (c) Silkie chicken. (TIF) [file pone.0173147.s003.tif]

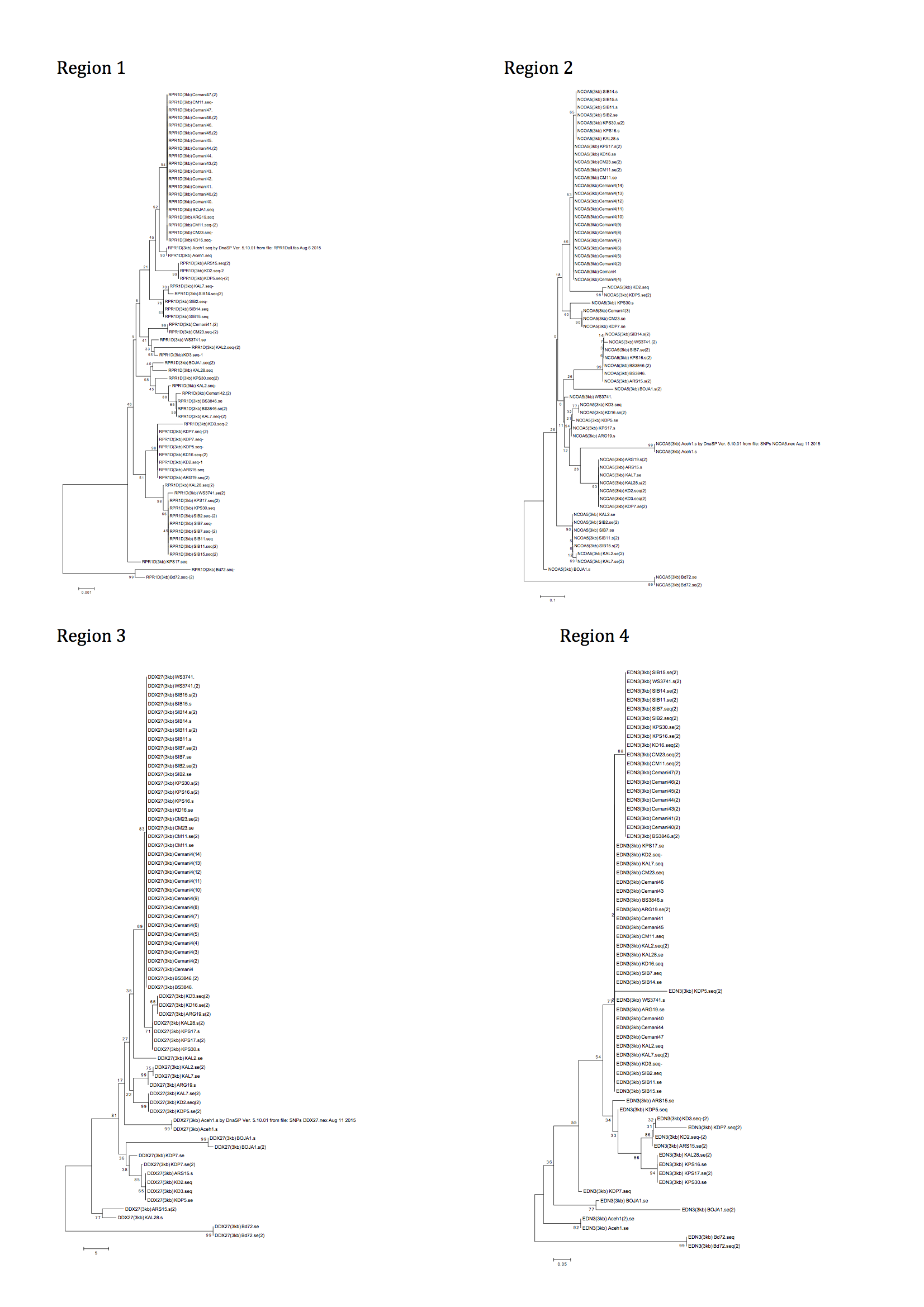

Supplement: S4 Fig — The phylogenetic relationship differs greatly from region to region. Two GJF haplotype sequences were used as outgroups. (TIF) [file pone.0173147.s004.tif]

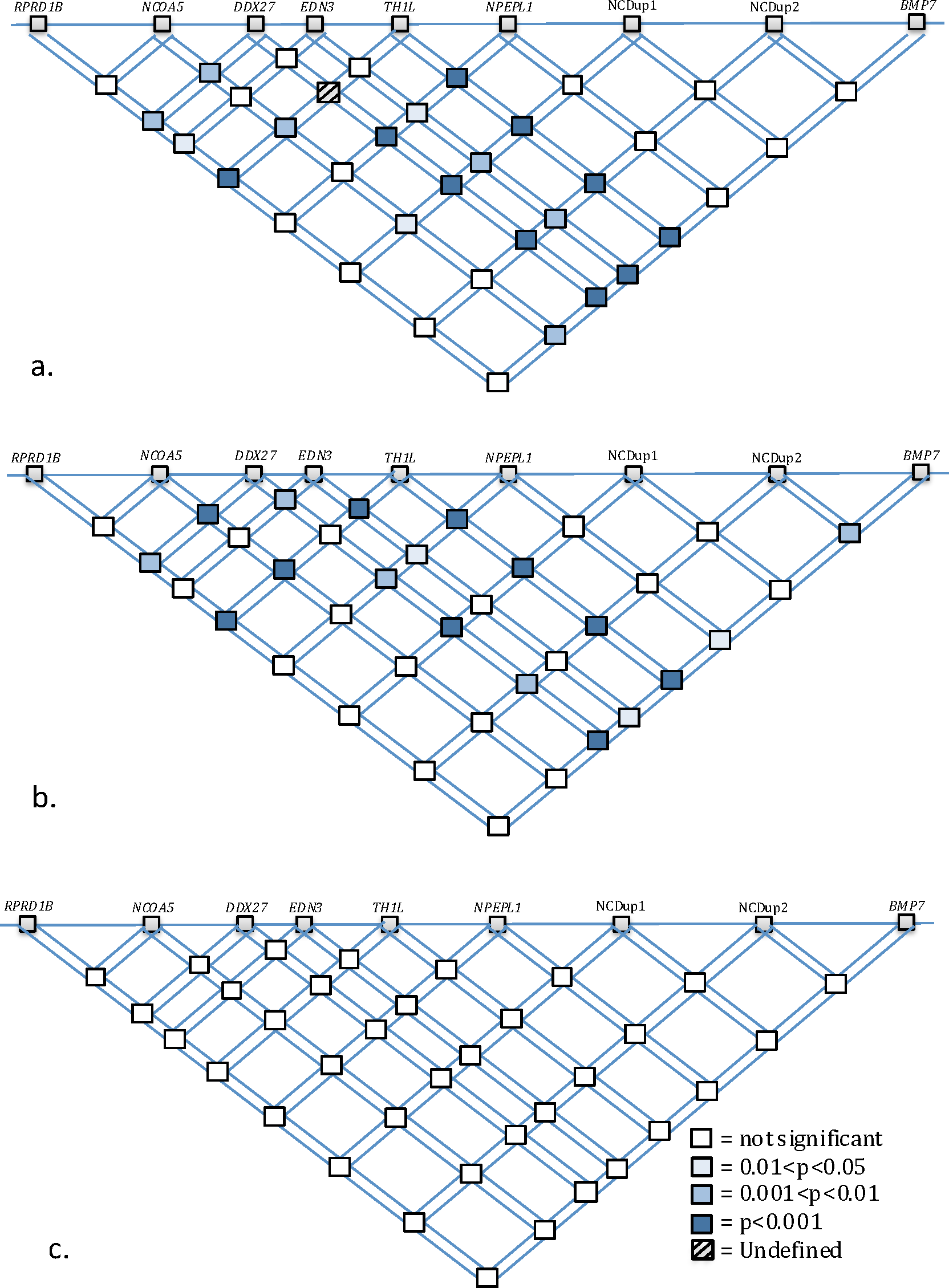

Supplement: S5 Fig — Results of the HKA test in each of the nine regions of Cemani (a), Silkie (b), and other domesticated chickens (c). The significant reduction in DNA polymorphism is found in Cemani and Silkie only for DDX27 in region 3, EDN3 in region 4, and TH1L in region 5 are compared. (TIF) [file pone.0173147.s005.tif]

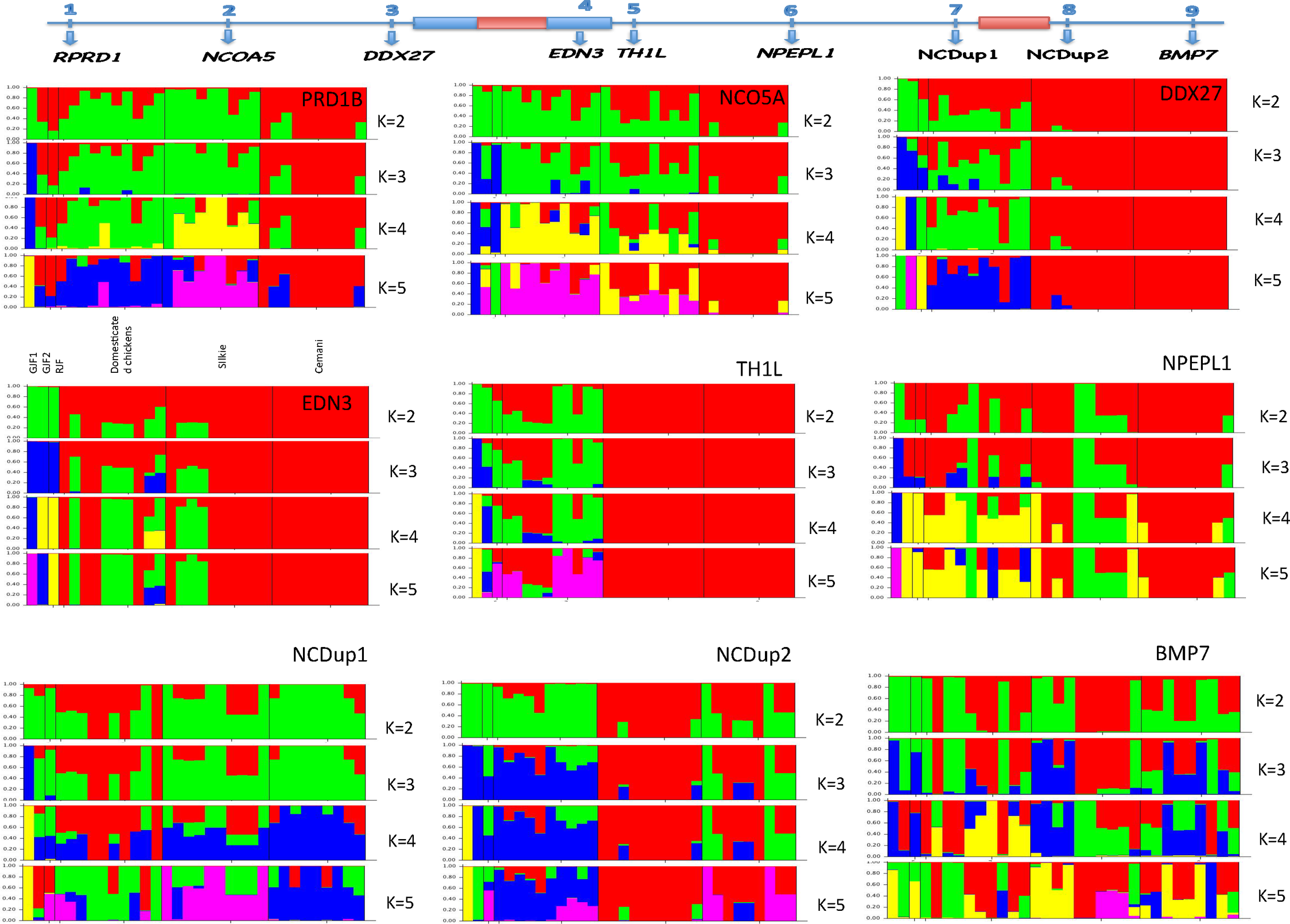

Supplement: S6 Fig — For regions 3–5, Cemani and Silkie exhibit nearly identical genetic components, whereas in other regions, there are no noticeable structural differences among chicken breeds and RJF. (TIF) [file pone.0173147.s006.tif]

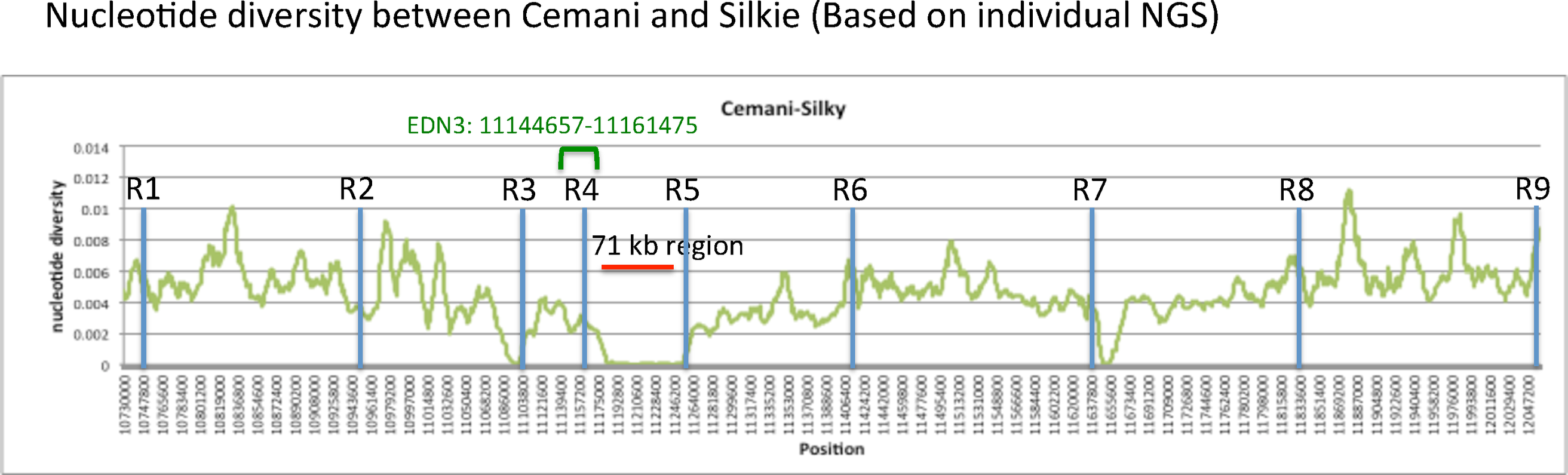

Supplement: S7 Fig — Bars with R1–R9 indicate the positions of the nine regions. Green square parentheses indicate the position of EDN3, and a red bar indicates the 71.4-kb region with low divergence between the two breeds. (TIF) [file pone.0173147.s007.tif]
