# Supplementary material for: The origin and evolution of fibromelanosis in domesticated chickens: Genomic comparison of Indonesian Cemani and Chinese Silkie breeds
Source: PLoS One. 2017 Apr 5;12(4):e0173147. doi: 10.1371/journal.pone.0173147 (PMC5381777; doi:10.1371/journal.pone.0173147)
Supplement: S1 Table — (PDF) [file pone.0173147.s008.pdf]

Primer of nine region surrounding duplicated region			
Primer name	Forward	Reverse	Position
RPRD1B (Region1)	5'-GCACACACTCAAACTACGAGGTTACAGT-3'	5'-GCTCAGTATCATTGGCTGTTAGATGGT-3'	10740207 - 10743372
NCO5 (Region 2)	5'-TGAGTTATGCTAGGAAGGTCAGGCTTCT-3'	5'-GGCATATGGCTCTCTGAGGAAAACAC-3'	10955102 - 10958453
DDX27 (Region 3)	5'-CTACCACGCTGGATGAGAAGATTGAGA-3'	5'-GTGCAGCCACATCTGTAGCTACCAAA-3'	11100827 - 11104025
EDN3 (Region 4)	5’-AATCGTGTCTATGGGAACGGTAAACG-3’	5’-TCGCTCTGTCCATCCAATTGTACTGAT-3’	11156158 - 11159283
TH1L (Region 5)	5'-GAGTTCTGCGTACATCTCTGGCAACA-3'	5'-TCATCCTTGTTTATGCTCACTCTCTTGTTC-3'	11249249 - 11252418
NPEPL1 (Region 6)	5'-GTGCCTTCAAAGCCACTGTAAAGCA-3’	5'-CCTGCCAGCTTTAACACAAGCCTTT-3	11409018 – 11412144
NCdup (Region 7)	5'-GACAATACGTCCAGCATCTCAGTTTACC-3'	5'-CCTACATGCACCCATTAGCTTGAAGA-3'	11637526 – 11640822
NCdup2 (Region 8)	5'-CTCTCGTCCACATTGAAATGAATCAAG-3'	5'-TCCCATCTCAGTGTCTATGAGAGCAGTT-3'	11831272 - 11834623
BMP7 (Region 9)	5'-GGTGTTTGACATCACTGCAACCAGTAAT-3'	5'-GCTTTGCCCTTAACAACAACACAGCAT-3’	12059056 - 12063056

Primer list between region 2-3 and region 5-6			
**Region 2 - 3**			
Primer name	Forward	Reverse	Position
D(2-3)	5'-AGAGTTGCAGGTAAGAAAGCTATG-3'	5'-AAGCTGACAAATTGCCTGAAG-3'	11036545-11037716
F(2-3)	5'-TTGTGCTGTTCAGTCAGGTTCT-3'	5'-TCAGCCTTCTGCATAAGTATCTGT-3'	11074442-11075542
**Region 5 - 6**			
Primer name	Forward	Reverse	
C(5-6)	5'-ACCTTATTAACCCATGGCATGT-3'	5'-ATAATGCTACGACAGCAGGAAAC-3'	11310054-11311124
D(5-6)	5'-TTTCTTGCTCAGGTTTGCAGTA-3'	5'-CAATTAGGTGAAGGCAGAATACAG-3'	11329264-11330394
E(5-6)	5'-AGGCATTCCTCACTTTAAGCAT-3'	5'-CACCTGTACTGATGTGCTTTGAG-3'	11345368-11346407
G(5-6)	5'-AACAAAGCTGGTTTGGTTCTG-3'	5'-TGCTTGTCTGACTCCTCTGTATG-3'	11384999-11386123
**Primer list for haplotype of *EDN3* variation and qPCR analysis**			
Primer name	Forward	Reverse	
AS044 (Shinomiya et al.2011)	5’-CCCAGCCTTCATTTCGGTGC-3’	5’-CCCTCCAAGCTCTGCTACTG-3’	11146917-11148035
qAS044	5’-CCTCATGTCTCGTAGCATAGGCTAACTC-3’	5’-TGACTTTATCACCATGTTTGAGCTTTCTC-3’	11147700-11147839
q46	5’-ACACCTCCACCACCCAAGAAT-3’	5’-GCGAATGGAGAATGAACAACATCA-3’	
**Primer list for duplication boundary detection (Dorshort et al. 2010)**			
Primer set name	Forward	Reverse	
A1	232 (AGAAACAAGGGTCAAGGTGAGC)	234 (TGGATCATTGGAGGAAGTGTTG)	
A2	200 (GGGATGGCTCTCACATAAAAGG)	234 (TGGATCATTGGAGGAAGTGTTG)	
B1	201(CTTGGCTCAGA T A TTCGCCTCT)	202 (AGGCACAGTCTGGCACATTAAA)	
B2	197 (GCAGCCTTT A TT A TTGCGTGTG)	201(CTTGGCTCAGA T A TTCGCCTCT)	
