# Supplementary material for: The origin and evolution of fibromelanosis in domesticated chickens: Genomic comparison of Indonesian Cemani and Chinese Silkie breeds
Source: PLoS One. 2017 Apr 5;12(4):e0173147. doi: 10.1371/journal.pone.0173147 (PMC5381777; doi:10.1371/journal.pone.0173147)
Supplement: S2 Table — (PDF) [file pone.0173147.s009.pdf]

Table S4 Reaction mixture and condition for PCR

	PCR1	PCR2	PCR3	PCR4
Total reaction volume (μl)	30	25	20	30
Taq polymerase	(Takara LA Taq™) 0.5 U/μl	(Takara ex Taq) 0.5 U/μl	SYBR® Premix Ex Taq™ II (Tli RNaseH Plus)	(Takara ex Taq) 0.5 U/μl
2.5mdNTP	3 μl	2.5 μl	SYBR Premix 10μl	3 μl
10 X buffer	(plus Mg^{2+}) 3μl	2.5 μl		(plus Mg^{2+}) 3 μl
10pM of forward and reverse primer	3μl	2.5 μl	1.6μl	3 μl
template	40 ng	10 ~ 100 ng	40 ng	20-100 ng
Reaction condition	D (denaturation) at 94 ºC for 3min.<br>30 cycles of D at 94 ºC for 3min., A(annealing) at 63~65 ºC for 1 min., and E(extenstion) at 72 ºC for 1min.<br>Final E at 72 ºC for 5min.	D at 95ºC for 5min.<br>30 cycles of D at 95 ºC for 30 sec., A at 58 ºC for 30 sec., and E at 72 ºC for 1min.<br>Final E at 72 ºC for 5min.	D at 95 ºC 1min.<br>40 cycles of D at 95°C for 5 sec. A/E at 60°C for 30 sec.	D at 94 ºC for 5min.<br>35 cycles of D at 94ºC for 30 sec., A at 58 ºC for 30 sec., and E at 72 ºC for 1min.<br>Final E at 72 ºC for 5min.
