# Supplementary material for: The origin and evolution of fibromelanosis in domesticated chickens: Genomic comparison of Indonesian Cemani and Chinese Silkie breeds
Source: PLoS One. 2017 Apr 5;12(4):e0173147. doi: 10.1371/journal.pone.0173147 (PMC5381777; doi:10.1371/journal.pone.0173147)
Supplement: S3 Table — (PDF) [file pone.0173147.s010.pdf]

Segregating sites in 12 *EDN3* haplotypes

**Site position**[*1]	**48**	**56**	**124**	**125**	**126**	**127**	**131**	**173**	**234**	**261**	**266**	**269**	**318**	**361**	**365**	**432**	**436**	**456**	**492**	**616**	**712**	**732**	**744**	**764**	**784**	**808**	**857**	**884**	**930**	**1013**	**1065**	**1066**	**1067**	**1094**	**1101**	**No.**[*2]
Haplotype 1	A	C	C	T	G	A	C	T	A	G	C	A	G	A	-	G	C	G	A	G	C	A	A	T	A	G	C	A	T	A	A	C	A	G	C	1
Haplotype 2	.	.	.	.	.	.	.	.	.	.	.	.	.	T	G	T	T	.	.	.	.	.	.	.	.	.	.	.	.	.	.	.	.	.	.	17
Haplotype 2’	.	.	.	.	.	.	.	.	.	.	.	.	.	T	G	T	T	.	.	.	.	.	.	.	G	.	.	.	.	.	.	.	.	.	.	1
Haplotype 3	.	.	.	.	.	.	.	.	.	.	.	.	.	.	-	.	.	.	.	C	.	T	.	C	.	A	T	.	.	G	.	.	.	.	.	1
Haplotype 4	.	.	.	.	.	.	.	.	.	.	.	.	.	T	G	.	.	.	.	.	.	.	.	.	.	.	.	.	.	.	.	.	.	.	.	23
Haplotype 5	G	T	T	-	-	-	T	.	.	.	T	C	A	.	-	.	.	.	G	.	.	.	G	C	.	.	.	.	.	.	.	.	.	A	T	2
Haplotype 6	.	T	.	.	.	.	.	C	.	.	.	.	A	.	-	T	T	A	G	C	A	.	.	C	.	.	.	.	.	G	.	.	.	-	-	2
Haplotype 7	.	.	.	.	.	.	.	.	.	.	.	.	.	.	-	.	.	.	G	C	.	T	.	C	.	A	.	.	.	G	.	.	.	.	.	2
Haplotype 8	.	T	T	.	.	.	T	.	G	.	T	C	A	.	-	.	.	.	G	.	.	.	G	C	.	.	.	G	G	.	.	.	.	.	.	2
Haplotype 9	.	.	.	.	.	.	.	.	.	.	.	.	.	T	G	.	.	.	.	.	.	.	.	.	.	.	.	.	.	.	-	-	-	.	.	1
Haplotype 10	.	.	.	.	.	.	.	.	.	.	.	.	.	T	G	.	.	.	G	.	.	.	.	.	.	.	.	.	.	.	.	.	.	.	.	2
Haplotype 11	.	T	.	.	.	.	.	C	.	A	.	.	A	.	-	.	.	A	G	.	.	.	.	C	.	.	.	.	.	.	.	.	.	.	.	1

The top row shows relative positions of segregating sites in the cloned segment. A shadowed cell indicates 1-bp deletion.

*1: Site position 1 corresponds to 11005500 in the reference sequence (NC_006107).

*2: No. of individuals observed in the sample of size of 17 homozygotes and 19 heterozygotes.
