# Supplementary material for: The origin and evolution of fibromelanosis in domesticated chickens: Genomic comparison of Indonesian Cemani and Chinese Silkie breeds
Source: PLoS One. 2017 Apr 5;12(4):e0173147. doi: 10.1371/journal.pone.0173147 (PMC5381777; doi:10.1371/journal.pone.0173147)
Supplement: S4 Table — The region ranges from nt 11,183,600 to 11,255,000 and includes part of DR1. Insertions and deletions are excluded. The colored columns indicate the Silkie (green)- or Cemani (red)-specific mutations. (PDF) [file pone.0173147.s011.pdf]

position	reference	Cemani	genotype	Silkie	genotype	L2 Taiwanese	genotype
11184107	T	T/C	het	T/C	het	T	hom
11184380	C	C/T	het	C/T	het	C	hom
11184505	C	C/G	het	C/G	het	C	hom
11184591	C	C/A	het	C/A	het	C	hom
11184672	A	A	hom	A/G	het	A	hom
11184694	G	G/A	het	G/A	het	G	hom
11184727	T	T/G	het	T/G	het	T	hom
11184734	A	A/T	het	A/T	het	A	hom
11185151	C	C/A	het	C/A	het	C	hom
11185935	C	C/T	het	C/T	het	C	hom
11186109	C	C/T	het	C/T	het	C	hom
11186125	T	T/C	het	T/C	het	T	hom
11186218	G	G/A	het	G/A	het	G	hom
11186220	G	G/A	het	G/A	het	G	hom
11186266	T	T/G	het	T/G	het	T	hom
11186267	G	G/T	het	G/T	het	G	hom
11186672	C	C/T	het	C/T	het	C	hom
11186808	G	G/A	het	G/A	het	G	hom
11187282	T	T/C	het	T/C	het	T	hom
11187334	C	C/T	het	C/T	het	C	hom
11187494	G	A	hom	A	hom	G	hom
11187816	G	G/A	het	G/A	het	G	hom
11187964	T	T/C	het	T/C	het	T	hom
11187996	T	T/C	het	T/C	het	T	hom
11188298	C	C/T	het	C/T	het	C	hom
11188585	A	A/G	het	A/G	het	A	hom
11188777	G	G/T	het	G/T	het	G	hom
11188845	T	C	hom	C	hom	T	hom
11189939	A	A/G	het	A/G	het	A	hom
11190074	A	A/G	het	A/G	het	A	hom
11190475	T	T/C	het	T/C	het	T	hom
11190652	T	T/C	het	T/C	het	T	hom
11190958	T	T/A	het	T/A	het	G	hom
11190958	T	T/G	het	T/G	het	T	hom
11191533	A	A/G	het	A/G	het	A	hom
11191638	A	A/G	het	A/G	het	A	hom
11191752	C	C/T	het	C/T	het	C	hom
11192379	T	T/C	het	T/C	het	T	hom
11192412	C	C/T	het	C/T	het	C	hom
11192860	G	G/A	het	G/A	het	G	hom
11193080	G	G/C	het	G/C	het	G	hom
11193105	A	A/G	het	A/G	het	A	hom
11193454	G	G/A	het	G/A	het	G	hom
11193610	T	C	hom	C	hom	T	hom
11193636	C	A	hom	A	hom	C	hom
11193723	G	G/A	het	G/A	het	G	hom
11193749	T	T/G	het	T/G	het	T	hom
11194503	T	T/A	het	T/A	het	T	hom
11194937	A	A/G	het	A/G	het	A	hom
11196053	T	T/A	het	T	hom	T	hom
11196105	A	A/G	het	A/G	het	A	hom
11197355	A	A/C	het	A/C	het	A	hom
11197426	T	T/C	het	T/C	het	T	hom
11201894	C	A	hom	A	hom	C	hom
11202063	G	A	hom	A	hom	G	hom

# of het_Cemani = 50
# of het_Silkie = 51
# of hom_Cemani = 106
# of hom_Silkie = 105

11202703	T	G	hom	T	hom	T	hom
11203349	C	T	hom	T	hom	C/G	het
11203758	C	T	hom	T	hom	C	hom
11204021	C	A	hom	A	hom	C	hom
11204052	C	T	hom	T	hom	C	hom
11204053	A	G	hom	G	hom	A	hom
11204131	C	A	hom	A	hom	C	hom
11204145	T	C	hom	C	hom	T	hom
11204268	C	T	hom	T	hom	C	hom
11204289	G	A	hom	A	hom	G	hom
11204553	A	G	hom	G	hom	A	hom
11205462	G	A	hom	A	hom	G	hom
11205485	C	T	hom	T	hom	C	hom
11205829	C	A	hom	A	hom	C	hom
11205845	T	C	hom	C	hom	T	hom
11205892	T	G	hom	G	hom	T	hom
11206338	A	C	hom	C	hom	A	hom
11206824	A	G	hom	G	hom	A	hom
11206998	G	T	hom	T	hom	G	hom
11207428	G	T	hom	T	hom	G	hom
11207467	C	T	hom	T	hom	C	hom
11207724	T	C	hom	C	hom	T	hom
11207940	G	C	hom	C	hom	G	hom
11208255	G	C	hom	C	hom	G	hom
11208366	G	C	hom	C	hom	G	hom
11209096	C	G	hom	G	hom	C	hom
11209342	G	A	hom	A	hom	G	hom
11209633	G	A	hom	A	hom	G	hom
11209692	G	G/A	het	G/A	het	G	hom
11210453	C	A	hom	A	hom	C	hom
11213748	C	T	hom	T	hom	C	hom
11213786	C	T	hom	T	hom	C	hom
11214040	T	A	hom	A	hom	T	hom
11214913	C	A	hom	A	hom	C	hom
11214923	A	G	hom	G	hom	A	hom
11215207	C	G	hom	G	hom	C	hom
11215871	G	A	hom	A	hom	G	hom
11217777	C	T	hom	T	hom	C	hom
11217989	C	T	hom	T	hom	C	hom
11218209	G	A	hom	A	hom	G	hom
11218613	T	A	hom	A	hom	T	hom
11219048	G	A	hom	A	hom	G	hom
11219232	G	T	hom	T	hom	G	hom
11219370	T	C	hom	C	hom	T	hom
11220573	G	C	hom	C	hom	G	hom
11220623	C	T	hom	T	hom	C	hom
11221946	G	A	hom	A	hom	G	hom
11222140	G	A	hom	A	hom	G	hom
11222666	C	A	hom	A	hom	C	hom
11222766	C	G	hom	G	hom	C	hom
11223603	A	G	hom	G	hom	A	hom
11224007	G	T	hom	T	hom	G	hom
11224077	A	G	hom	G	hom	A	hom
11224374	C	A	hom	A	hom	C	hom
11224518	C	A	hom	A	hom	C	hom
11224519	T	C	hom	C	hom	T	hom

11224682	A	G	hom	G	hom	A	hom
11224974	C	G	hom	G	hom	C	hom
11225127	C	T	hom	T	hom	C	hom
11225316	A	G	hom	G	hom	A	hom
11226108	T	C	hom	C	hom	T	hom
11230298	T	C	hom	C	hom	T	hom
11231669	A	C	hom	C	hom	A	hom
11232740	A	G	hom	G	hom	A	hom
11232802	A	C	hom	C	hom	A	hom
11233294	A	G	hom	G	hom	A	hom
11233373	G	T	hom	T	hom	G	hom
11233533	C	G	hom	G	hom	C	hom
11233849	G	C	hom	C	hom	G	hom
11234218	C	T	hom	T	hom	C	hom
11234374	G	A	hom	A	hom	G	hom
11234810	T	T/G	het	T/G	het	T	hom
11234814	G	T	hom	T	hom	G	hom
11234820	A	T	hom	T	hom	A	hom
11235522	C	T	hom	T	hom	C	hom
11235847	T	G	hom	G	hom	T	hom
11235959	T	C	hom	C	hom	T	hom
11236606	C	C		C/T	het	C	hom
11237812	T	C	hom	C	hom	T	hom
11237813	T	G	hom	G	hom	T	hom
11238450	G	A	hom	A	hom	G	hom
11238851	C	T	hom	T	hom	C	hom
11239021	C	T	hom	T	hom	C	hom
11239313	T	G	hom	G	hom	T	hom
11239899	C	G	hom	G	hom	C	hom
11240721	G	A	hom	A	hom	G	hom
11241229	G	C	hom	C	hom	G	hom
11241403	A	G	hom	G	hom	A	hom
11241417	C	T	hom	T	hom	C	hom
11244911	A	C	hom	C	hom	A	hom
11244955	C	T	hom	T	hom	C	hom
11245067	T	C	hom	C	hom	T	hom
11245293	T	C	hom	C	hom	T	hom
11247151	C	T	hom	T	hom	C	hom
11248936	G	A	hom	A	hom	G	hom
11249067	C	T	hom	T	hom	C	hom
11249122	T	C	hom	C	hom	T	hom
11250412	G	A	hom	A	hom	G	hom
11251087	A	G	hom	G	hom	A	hom
11251132	G	C	hom	C	hom	G	hom
11251383	C	A	hom	A	hom	C	hom
11254430	C	T	hom	T	hom	C	hom
11254491	G	A	hom	A	hom	G	hom
